# Computational prognostic evaluation of Alzheimer’s drugs from FDA-approved database through structural conformational dynamics and drug repositioning approaches

**DOI:** 10.1038/s41598-023-45347-1

**Published:** 2023-10-21

**Authors:** Mubashir Hassan, Saba Shahzadi, Muhammad Yasir, Wanjoo Chun, Andrzej Kloczkowski

**Affiliations:** 1 The Steve and Cindy Rasmussen Institute for Genomic Medicine, Nationwide Children’s Hospital, Columbus, OH 43205 USA; 2 Department of Pharmacology, College of Medicine, Kangwon National University, Chuncheon, South Korea; 3 Department of Pediatrics, The Ohio State University, Columbus, OH 43205 USA

**Keywords:** Biophysics, Computational biology and bioinformatics, Drug discovery, Neuroscience

## Abstract

Drug designing is high-priced and time taking process with low success rate. To overcome this obligation, computational drug repositioning technique is being promptly used to predict the possible therapeutic effects of FDA approved drugs against multiple diseases. In this computational study, protein modeling, shape-based screening, molecular docking, pharmacogenomics, and molecular dynamic simulation approaches have been utilized to retrieve the FDA approved drugs against AD. The predicted MADD protein structure was designed by homology modeling and characterized through different computational resources. Donepezil and galantamine were implanted as standard drugs and drugs were screened out based on structural similarities. Furthermore, these drugs were evaluated and based on binding energy (Kcal/mol) profiles against MADD through PyRx tool. Moreover, pharmacogenomics analysis showed good possible associations with AD mediated genes and confirmed through detail literature survey. The best 6 drug (darifenacin, astemizole, tubocurarine, elacridar, sertindole and tariquidar) further docked and analyzed their interaction behavior through hydrogen binding. Finally, MD simulation study were carried out on these drugs and evaluated their stability behavior by generating root mean square deviation and fluctuations (RMSD/F), radius of gyration (Rg) and soluble accessible surface area (SASA) graphs. Taken together, darifenacin, astemizole, tubocurarine, elacridar, sertindole and tariquidar displayed good lead like profile as compared with standard and can be used as possible therapeutic agent in the treatment of AD after in-vitro and in-vivo assessment.

## Introduction

Alzheimer’s disease (AD), a neurodegenerative disorder usually characterized by memory loss in older persons^1,2^. Globally, it has been observed that around 35 million people are affected by AD annually and this number is believed to double in next 20 years. In USA, 6.7 million American are living with dementia having age greater than 65^3^. The etiology of AD is highly complex, however, there are some biomarkers including low levels of acetylcholine, β-amyloid (Aβ) deposits, tau-protein aggregation, neurofibrillary tangles (NFTs), oxidative stress, and dys-homeostasis of bio-metals are used for the diagnosis of AD^2,4^. These available clinical therapeutics show only partial effectiveness in ameliorating the AD symptoms^5^.

Drug development is time consuming and overpriced process however, computational techniques particularly drug repurposing is being used in the present time^6,7^. Drug repositioning is basic computational approach to predict the therapeutic potential of known drugs against different disease by targeting different receptors^8,9^. However, in present time, medical research emphases on the factors that are thought to contribute to AD development, such as tau proteins and Aβ deposits^10^. MAP kinase-activating death domain (MADD) protein is bulky a protein comprises 1647 amino acids and four different domains such as uDENN, cDENN, dDENN and death domain, respectively. Furthermore, six disordered regions are also present in MADD structure with different residues lengths. MADD protein plays a significant role in regulating cell proliferation, survival, and death through alternative mRNA splicing indifferent disease such as Deeah and AD^11^. MADD protein also links TNF receptor superfamily member 1A (TNFRSF1A) with MAP kinase activation and important for regulatory role in physiological cell death^12^.

In the present study, Food and Drug Administration (FDA)-approved drugs were screened against AD by taking double standards donepezil and galantamine through shape-based screening technique. The screened hits underwent molecular docking analysis using PyRx against MADD protein. Moreover, pharmacogenomics analysis was carried out and best drug interacting with AD-medicated genes were shortlisted from the pool of selected drugs. The best-selected drugs were further examined by a docking procedure with AutoDock to check their binding affinities against the MADD protein. Finally, the best-generated docked complexes were further analyzed through molecular dynamics simulations to observe the structural stability through RMSD, RMSF, Rg, and SASA graphs.

## Computational methodology

### Retrieval of MADD protein sequence

The amino acid sequence of MADD protein having accession number Q8WXG6 (https://www.uniprot.org/uniprot/Q8WXG6) was accessed from the UniProt database^13^. MADD protein contains four major domains (uDENN, cDENN, dDENN and Death domain) and unstructured regions..

### Modelling of MADD protein and domain assembly

The complete three-dimensional (3D) structure of MADD is not available in Protein Data Bank^14^ (PDB; https://www.rcsb.org/); therefore, homology modelling based method was used to predict the complete structure of MADD protein. There are different online protein prediction servers, however, trRosetta (transform-restrained Rosetta) is the most recent protein structure prediction server. trRosetta is a web-based platform for fast and accurate protein structure prediction, powered by deep learning and Rosetta (https://yanglab.nankai.edu.cn/trRosetta/). However, to predict the complete MADD protein at once using server, its much difficult therefore, all four domains (uDENN, cDENN, dDENN and Death domain) and unstructured/disordered regions were modelled separately using trRosetta. After modelling of all four domains and unstructured/disordered regions, The Domain Enhanced MOdeling (DEMO; https://zhanggroup.org/DEMO/) was used to assemble all structure using default parameters^15^. Demo is a method for automated assembly of full-length structural models of multi-domain proteins. Starting from individual domain structures, DEMO first identify quaternary structure templates that have similar component domains by domain-level structural alignments using TM-align. Replica-exchange Monte Carlo simulations are used to assemble full-length models, as guided by the inter-domain distance profiles collected from the top-ranked quaternary templates. The final models with the lowest energy are selected from Monte Carlo trajectories, followed by atomic-level refinements using fragment-guided MD simulations^15^.

### Structure analysis of MADD protein

The assemble model structure of MADD protein was analyzed using different computational approaches to check its stability behavior. To do this, initially Ramachandran Plot Server (https://zlab.umassmed.edu/bu/rama/index.pl) and MolProbity server (http://molprobity.biochem.duke.edu/) were used to check the amino acids and overall protein conformation with respect to phi (φ) and psi (ψ) angles by generating Ramachandran graph. The stability behavior of target protein is dependent upon the occurrence of residues in allowed and disallowed regions. Furthermore, the ProtParam tool was employed to predict their theoretical PIs, extinction coefficients, aliphatic and instability indexes, and GRAVY values^16^. The overall protein architecture and the statistical percentage values of α-helices, β-sheets, coils, and turns were retrieved from the online VADAR 1.8 server^17^. FRST serves to validate the energy of a protein structure^18^. The server computes both an overall and a per-residue energy profile of a protein structure. Furthermore, Quality Model Energy Analysis (QMEAN) was employed to further evaluate the model quality (https://swissmodel.expasy.org/qmean/). Another known model server SaliLab Model Evaluation Server (https://modbase.compbio.ucsf.edu/evaluation/) was employed to check the predicted model quality based on TSVMod and Modeller values. Finally, the modelled structure was further refine using GalaxyWEB (https://galaxy.seoklab.org/) before going to utilize it for virtual screening, molecular docking, and dynamic simulation analysis. The graphical representation of model structure was done using Discovery Studio 2.1 Client^19^, and UCSF Chimera X^20^. Furthermore, after prediction of protein model Split-Statistical Potentials Server (http://aleph.upf.edu/spserver/) was employed to identify the native conformation behavior and to evaluate the accuracy of protein folds^21^.

### Shape-based virtual screening of chemical scaffolds

The Swiss Similarity, an online platform that allows you to identify the similar chemical hits from Food and Drug Administration (FDA) and other libraries with respect to the reference template structure^22^. In our current research approach, couple of standard drugs; donepezil and galantamine are being used against AD^5,23,24^ were implanted and retrieved similar drugs hits based on structural similarity. The chemical structures of donepezil and galantamine were retrieved from Drug Bank (https://go.drugbank.com/) having accession numbers (DB00843 & DB00674) and used as template molecule to screen FDA-approved drugs. All the screened drugs were ranked according to their predicted similarity scoring values. The best screened drugs were sketched in ACD/ChemSketch and utilized for molecular docking experiments.

### Prediction of active binding sites of MADD protein

The Prankweb (http://prankweb.cz/), an online source that explores the probability of amino acids involved in the formation of active binding sites. Prankweb is a template-free machine learning method based on the prediction of local chemical neighborhood ligandability centered on points placed on a solvent-accessible protein surface^25^. Points with a high ligandability score are then clustered to form the resulting ligand binding sites. The binding pocket information was not available in PDB; therefore, active binding sites residues of MADD protein were predicted by using Prankweb. The death domain is highly significant domain with functional association with AD^12^. Therefore, 3D modeled structure of death domain was utilized and predicts possible binding pockets with high probability values of amino acids^25^.

### Virtual screening using PyRx

Before conducting our docking experiments, all the screened drugs were sketched in ACD/ChemSketch tool and accessed in mol format. Furthermore, UCSF Chimera 1.10.1 tool was employed for energy minimization of each ligand having default parameters such as steepest descent and conjugate gradient steps 100 with step size 0.02 (Å), and update interval was fixed at 10. In PyRx docking experiment, all screened drugs were docked with death domain of MADD using default procedure^26^. Before, docking binding pocket of target protein was confirmed from Prankweb and literature data. In docking experiments, the grid box dimension values were adjusted as center_X = − 0.8961, Y = − 1.6716 and Z = 0.3732 whereas, size_X = 37.8273, Y = 36.5416 Z = 36.5756, respectively, with by default exhaustiveness = 8 value. The grid box size was adjusted on binding pocket residues to allows the ligand to move freely in the search space. Furthermore, the generated docked complexes were keenly analyzed to view their binding conformational poses at active binding site of MADD protein. Moreover, these docked complexes were evaluated based on the lowest binding energy (Kcal/mol) values and binding interaction pattern between ligands and target protein. The graphical depictions of all the docked complexes were accomplished by UCSF Chimera 1.10.1 and Discovery Studio (2.1.0), respectively.

Furthermore, another docking experiment was employed on best screened drugs against MADD protein using AutoDock 4.2 tool^27^. In brief, for receptor protein, the polar hydrogen atoms and Kollman charges were assigned. For ligand, Gasteiger partial charges were designated, and non-polar hydrogen atoms were merged. All the torsion angles for screened drugs were set free to rotate through the docking experiment. A grid map of 80 × 80 × 80 Å was adjusted on the binding pocket of MADD to generate the grid map and to get the best conformational state of docking. A total of 100 number of runs were adjusted using docking experiments. The Lamarckian genetic algorithm (LGA) and empirical free energy function were applied by taking docking parameters default^28^. All the docked complexes were further evaluated on lowest binding energy (kcal/mol) values and hydrogen and hydrophobic interactions analysis using Discovery Studio (2.1.0) and UCSF Chimera 1.10.1.

### Designing of pharmacogenomics networks

To design the pharmacogenomics network model for best-selected drugs, Drug Gene Interaction Databases (DGIdb) (https://www.dgidb.org/) and Drug Signatures Database (DSigDB) (http://dsigdb.tanlab.org/DSigDBv1.0/) were employed to obtain the possible list of different disease-associated genes. Furthermore, a detailed literature survey was performed against all predicted genes to identify its involvement in AD. Moreover, clumps of different diseases associated genes were sorted based on MADD and remaining disease-associated genes were eliminated from the dataset.

### Molecular dynamics (MD) simulations

The best screened drugs-complexes having good energy values were selected to understand the residual backbone flexibility of protein structure; MD simulations were carried out by Groningen Machine for Chemicals Simulations (GROMACS 4.5.4 package^29^, with GROMOS 96 force field^30^. The protein topology was designed by pdb2gmx command by employing GROMOS 96 force field. Moreover, simulation box with a minimum distance to any wall of 10 Å (1.0 nm) was generated on complex by editconf command. Moreover, box was filled with solvent molecules using gmx solvate command by employing spc216.gro water model. The overall system charge was neutralized by adding ions. The steepest descent approach (1000 ps) for protein structure was applied for energy minimization. For energy minimization the nsteps = 50,000 were adjusted with energy step size (emstep) 0.01 value. Particle Mesh Ewald (PME) method was employed for energy calculation and for electrostatic and van der waals interactions; cut-off distance for the short-range VdW (rvdw) was set to 14 Å, whereas neighbor list (rlist) and nstlist values were adjusted as 1.0 and 10, respectively, in em.mdp file^31^. This method permits the use of the Ewald summation at a computational cost comparable with that of a simple truncation method of 10 Å or less, and the linear constraint solver (LINCS)^32^ algorithm was used for covalent bond constraints and the time step was set to 0.002 ps. Finally, the molecular dynamics simulation was carried out at 100 ns with nsteps 50,000,000 in md.mdp file. Different structural evaluations such as root mean square deviations and fluctuations (RMSD/RMSF), solvent accessible surface areas (SASA) and radii of gyration (Rg) of back bone residues were analyzed through Xmgrace software (http://plasma-gate.weizmann.ac.il/Grace/).

## Results and discussion

**Figure 1:**
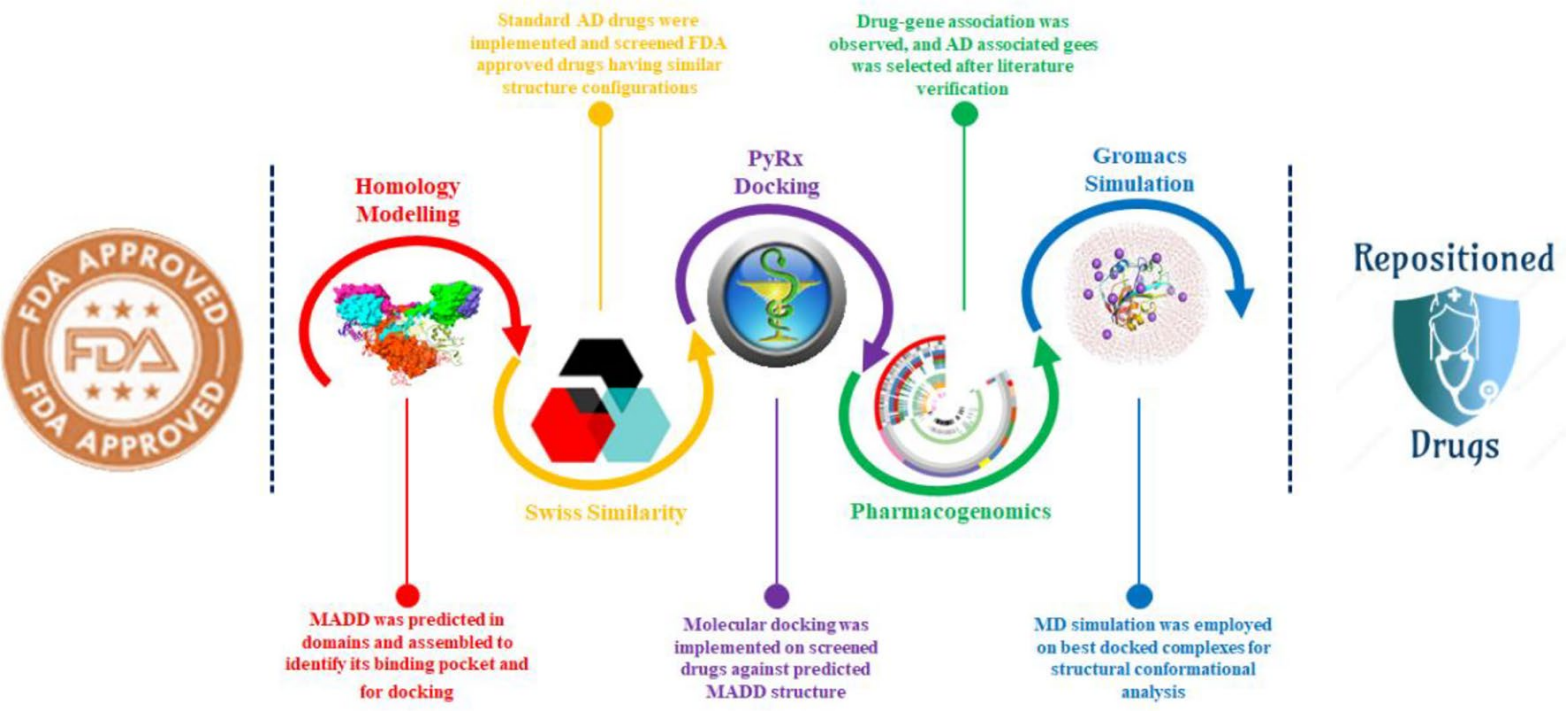
Proposed mechanistic flowchart of repositioned drugs.

The overall computational drug repositioning flowchart showed the multiple steps involved in this study (Fig. 1). A total of 631 drugs have been evaluated by employing different computational resources through PyRx screening, pharmacogenomics analysis, molecular docking, and MD simulations, respectively.

### Protparam analysis of MADD protein

ProtParam (https://web.expasy.org/protparam/) is an online tool which analyse the protein based on various physical and chemical parameters, respectively. The significant parameters include molecular weight (mg/mol), theoretical *pI*, amino acid composition, atomic composition, extinction coefficient, estimated half-life, instability/aliphatic indexes, and grand average of hydropathicity (GRAVY), respectively. The generated theoretical *pI* value of MADD protein is calculated by the accumulation of average isotopic masses and pK values of linear amino acids present in the proteins. The prior published research data depicted that proteins are distributed across a wide range of *pI* values (4.31–11.78)^33^. The MADD protein exhibited 5.49 *pI* value which is comparable with the standard values (4.31–11.78). Moreover, the aliphatic index value also showed the stability and relative volume occupied by aliphatic side chain residues. The predicted physiochemical properties showed the reliability, efficacy, and stability of the MADD protein. The predicted values of both instability and aliphatic indexes 58.81 and 74.96 are justifiable with standard values. The GRAVY value is the sum of hydropathy values of all residues in protein^34^. The prior research reports justified that the GRAVY negative and positive values show the hydrophilic and hydrophobic behavior of protein structure^33^. The negative GRAVY value (− 0.517) of MADD protein indicates the hydrophilic behavior (Table 1).

**Table 1:** The physiochemical properties of MADD protein.

Protein parameters	Predicted values
Number of amino acids	1165
Theoretical *PI*	5.49
Negatively charged residues (Asp + Glu)	157
Positively charged residues (Arg + Lys)	131
Total number of atoms	17,974
Instability index	58.81
Aliphatic index	74.96
GRAVY	− 0.517

### Structural assessment of MADD protein

**Figure 2:**
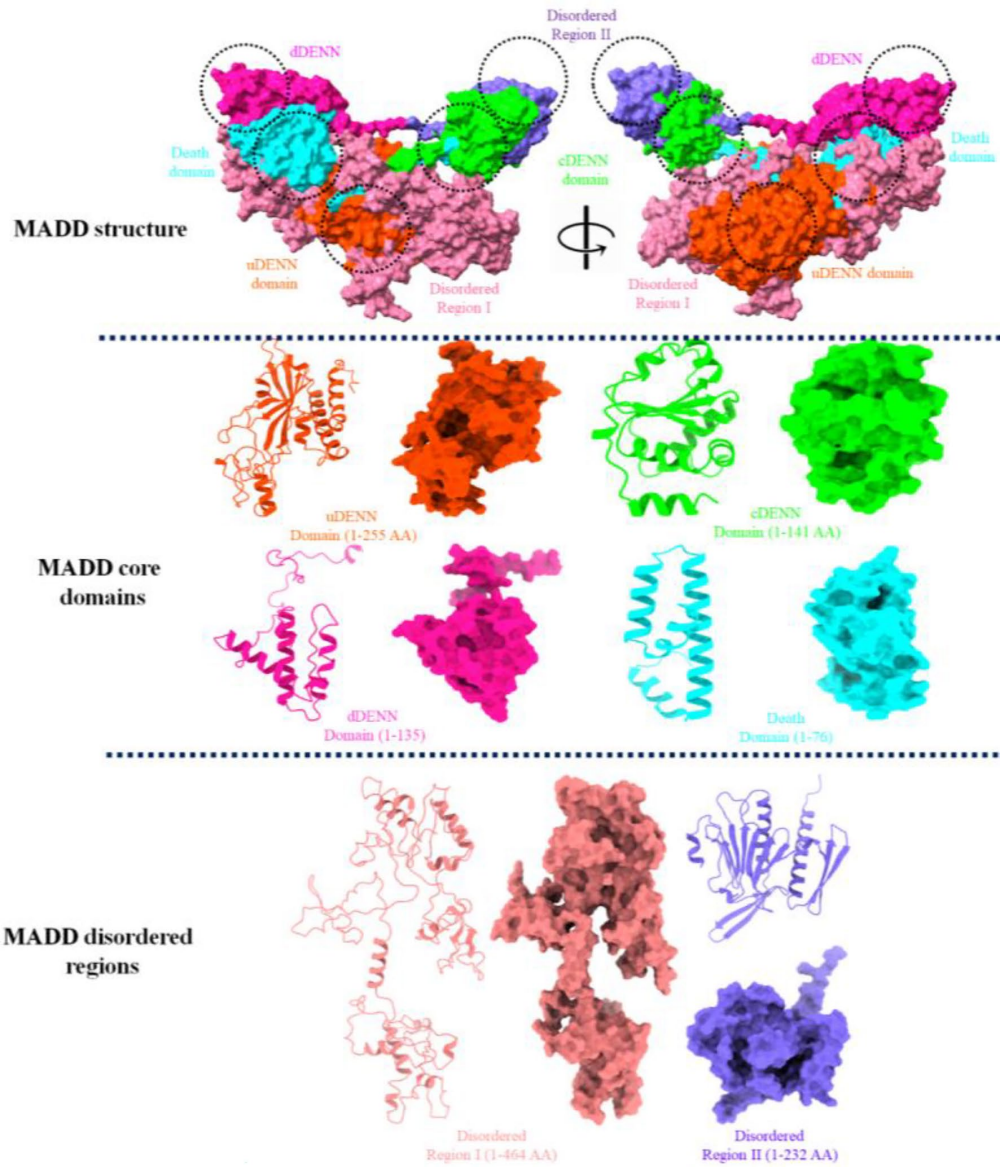
The overall protein structure of MADD protein. The figure showed the three main parts, the overall protein structure, its four core domains and couple of disordered regions.

MAP kinase-activating death domain (MADD) is a human protein encoded by *MADD* gene^35^. MADD, a bulky protein structure which comprises 1647 amino acids having molecular mass 183.303 KDa. The structural analysis showed that MADD contains four major domains such as uDENN (14-268 AA), cDENN (289-429 AA), dDENN (431-565 AA) and death domain (1340-1415 AA), respectively with disordered regions (https://www.uniprot.org/uniprot/Q8WXG6) (Fig. 2).

### VADAR analysis

VADAR is an online server which predict the protein volume, area, dihedral angle reports and statistical evaluation. The generated MADD model structure showed different architectures related to α-helices, β-sheets, coils and turn, respectively. The overall protein structure analysis showed that MADD consists of 25% α-helices, 19% β-sheets, 55% coils and 20% turns, respectively. Moreover, Ramachandran plots and values depicted that 92.68% of amino acids were present in favored region with good accuracy of phi (φ) and psi (ψ) angles which showed the good accuracy of our predicted model.

### Galaxy refine analysis

**Figure 3:**
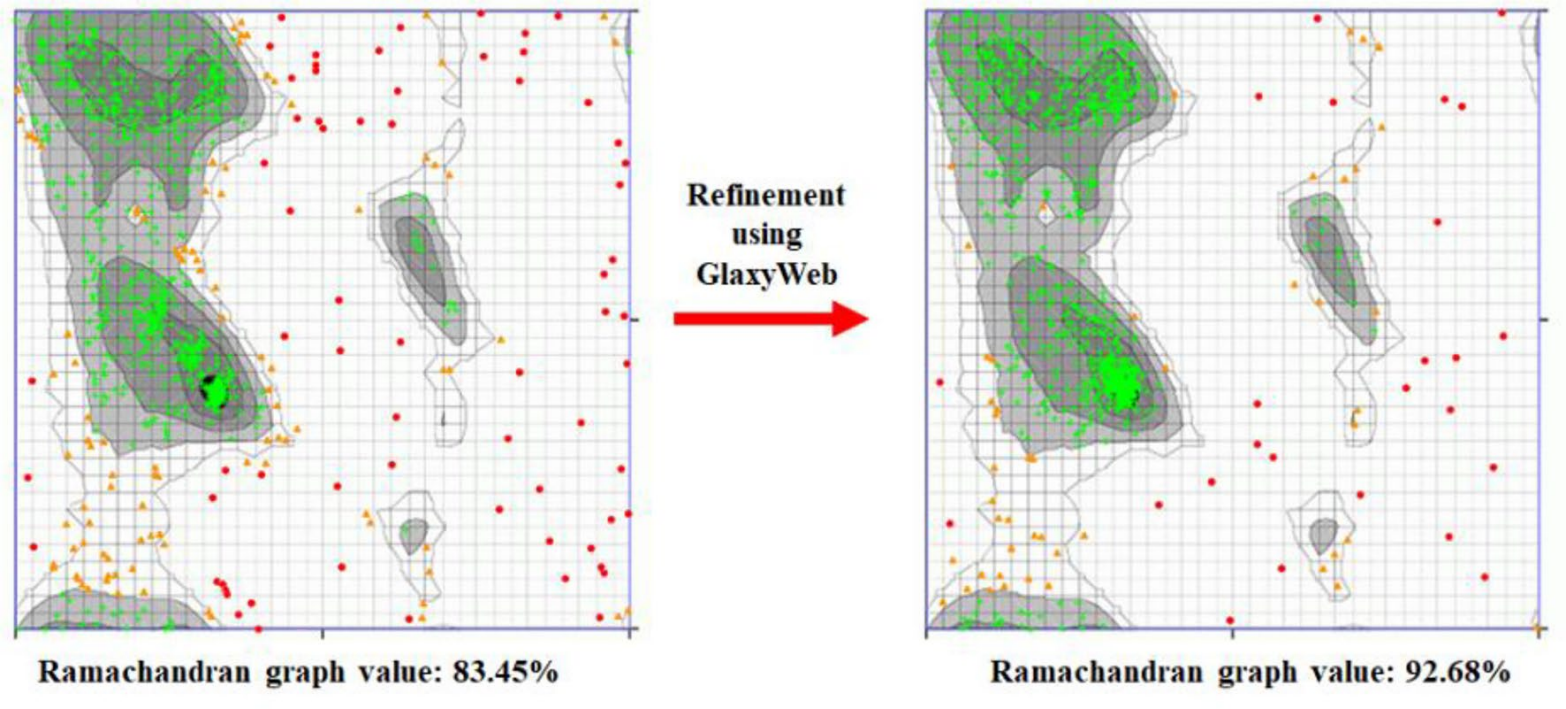
Ramachandran graphs of predicted MADD protein.

Galaxy-refine is an good source to resolve the ambiguities in the modelled structure based on different parameters such as GDT-HA, RMSD, MolProbity, clash score, poor rotamers and Rama favored, respectively^36^. The initial predicted structure of MADD protein depicted MolProbity: 3.809–2.871, clash score: 88.4–47.8, poor rotamers: 5.7–1.4 and Rama favored: 72.4–85.8, respectively whereas, the values have been rectified to improve the modelled MADD structure (Supplementary Table S1). Moreover, the online Ramachandran graph server (https://www.umassmed.edu/zlab/) generate both Ramachandran graphs which have been mentioned in Fig. 3. The comparative results showed that the Galaxy refine clearly improved the modelled MADD structure which can be further implied for structure and docking analysis.

### FRST energy validation against MADD

The energy validation of predicted MADD structure was analyzed using FRST evaluation tool based on overall and a per-residue energy profile of a protein structure. The FRST energy calculation is composed of rapdf pairwise potential, solvation potential, backbone hydrogen bonds and torsion angle potential and composite score values (http://protein.cribi.unipd.it/frst/). The rapdf potential^37^ is a distance-dependent residue-specific all-atom probability discriminatory function. Similarly, solvation potential to likelihood for each of 20 amino acids to adopt a given relative solvent accessibility is used to derive the pseudo-energy^38^. The torsion angle potential (TORS) is derived from the propensities of each amino acid to adopt different combinations of (phi, psi) torsion angles^39^. The total energy is again summed over all individual contributions in a protein. In our generated MADD results, rapdf, solvation, torsion and composite FRST energies (Kcal/mol) which showed the stability of model structure (Table 2).

**Table 2:** The energy evaluation of MADD protein.

Protein energy parameters	Predicted scores (Kcal/mol)
Rapdf energy	− 19,038.2
Solvation energy	9.04
Torsion energy	− 50.96
Composite FRST energy	− 60,911.62

### Quality model energy analysis

**Figure 4:**
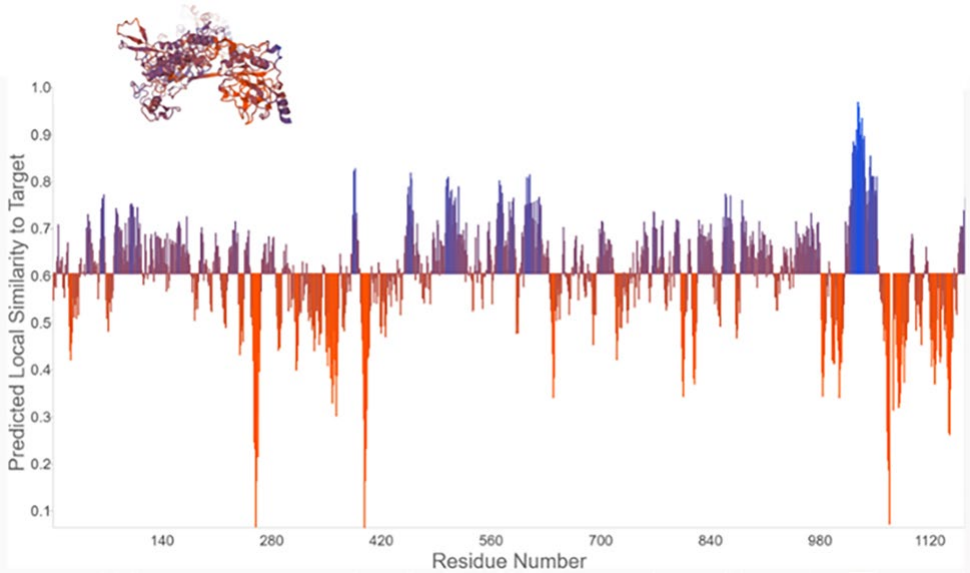
QMEAN Quality assessment of MADD protein.

Qualitative Model Energy ANalysis (QMEAN) is a composite scoring function which derive both global and local absolute quality estimates based on one single model (https://swissmodel.expasy.org/qmean/). The generated results showed that MADD protein exhibited − 6.64 value which is comparable with standard values (0,1). The prior data showed that different residues clumps showed lowest score value 0.1 which represents the quality assessment of predicted MADD model. However, most amino acids patches in the predicted model showed higher values > 0.6 depicts accurate and stable behavior of MADD protein. The blue peak showed highest prediction value having best quality results (> 0.8) of predicted model. Moreover, the purple color represents also showed good accuracy of MADD protein with scoring values range from 0.6 to 0.8. However, the orange values showed the de-stable behavior of MADD protein with values less than 0.6. The overall results showed that predicted MADD model exhibited good stable behavior and could be used for further analysis (Fig. 4).

### Native conformational behavior evaluation

**Figure 5:**
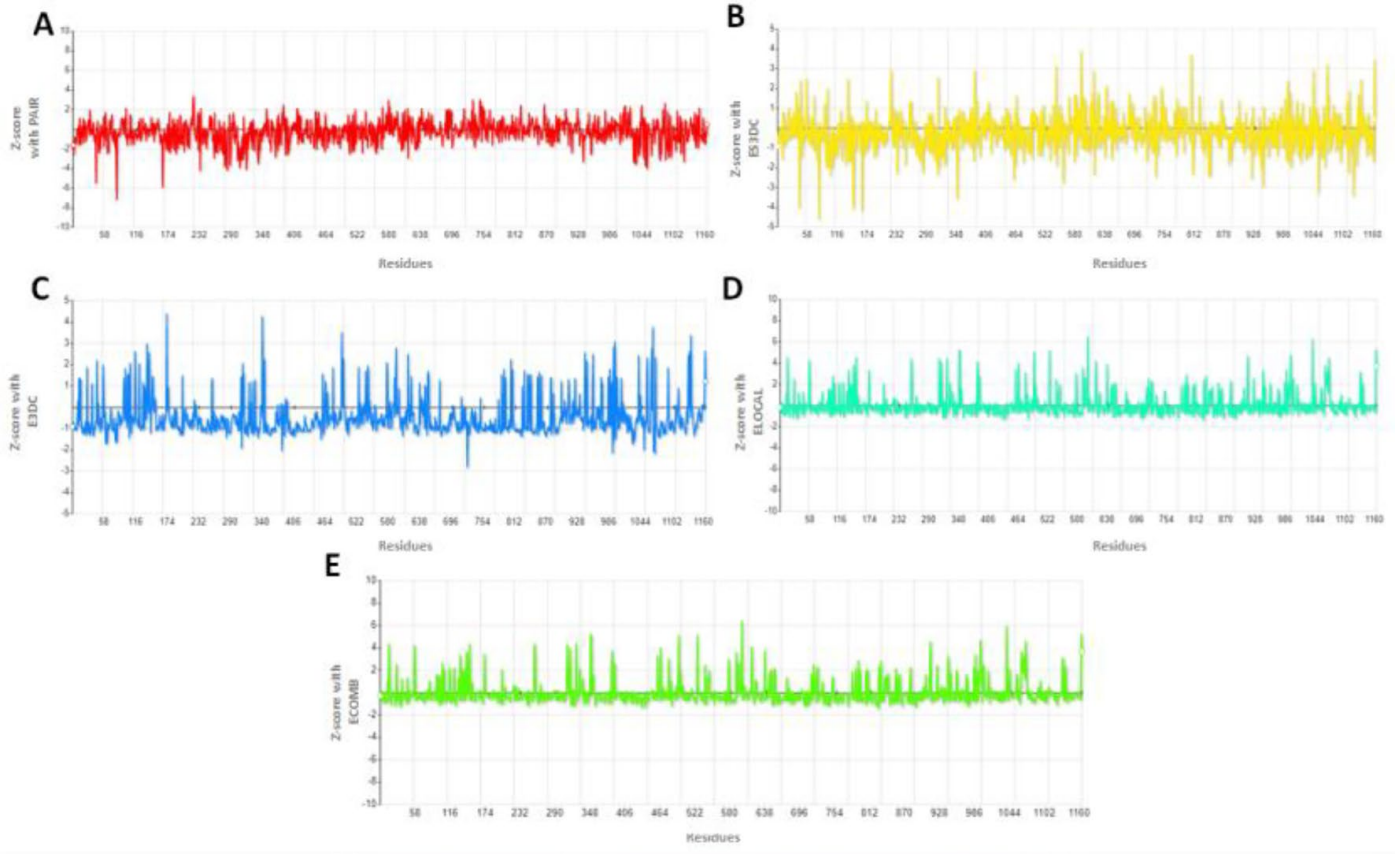
(**A**–**E**) SPS analysis of MADD protein to predict their folding and native conformation pattern.

After prediction the protein models, native conformation behavior is significant parameter is to evaluate the accuracy of protein folds. SPS server (http://aleph.upf.edu/spserver/index.php/init.html), is a knowledge-based scoring functions used for correct folding of proteins, stability of mutant proteins, and assess outcomes of docking experiments. The generated results were evaluated based on five different parameters such as PAIR, ES3DC, ECOMB, ELOCAL, and E3DC respectively. The PAIR and ES3DC parameters are focused on amino acid frequencies along distances and their environments such as hydrophobicity of each amino acid, solvent accessibility, and secondary structure, respectively. Figure 5A, B showed that residues fluctuations with respect to z-score values. In PAIR parameters analysis, residues showed stable frequency throughout the protein structure except amino acids at positions 58, 60, 174, 696 and 1100, respectively. The graph line remains stable at center value 0 with little deviations range from 2 and − 2. In ES3DC analysis in correlation with Z-score, the generated graph line (yellow) showed high fluctuations at the terminal part of MADD protein. However, the average value ranges from 3 to − 3 in the energy folding graph. Our results depicted that MADD protein exhibited hydrophobicity and solvent accessibility properties, respectively. Moreover, both E3DC and ELOCAL parameters are also implicated on MADD protein based on amino acid frequencies along distances of pairs referred by the hydrophobicity of the amino acids and the rest of their environments, whereas ECOMB is combinatorial scores of all three parameters such as ES3DC, ELOCAL and E3DC, respectively. Figure 5C displayed the residual fluctuations graph at different positions in the MADD structure whereas, ELOCAL parameter showed less fluctuations based on Z-score values (Fig. 5D). In combine effects, the ECOMB parameter exhibited stable behavior and good folding of model MADD protein. The fluctuation peaks were range from 0 to 3, however, at various position range raised up to 4 (Fig. 5E).

### Binding pocket analysis of MADD protein

**Figure 6:**
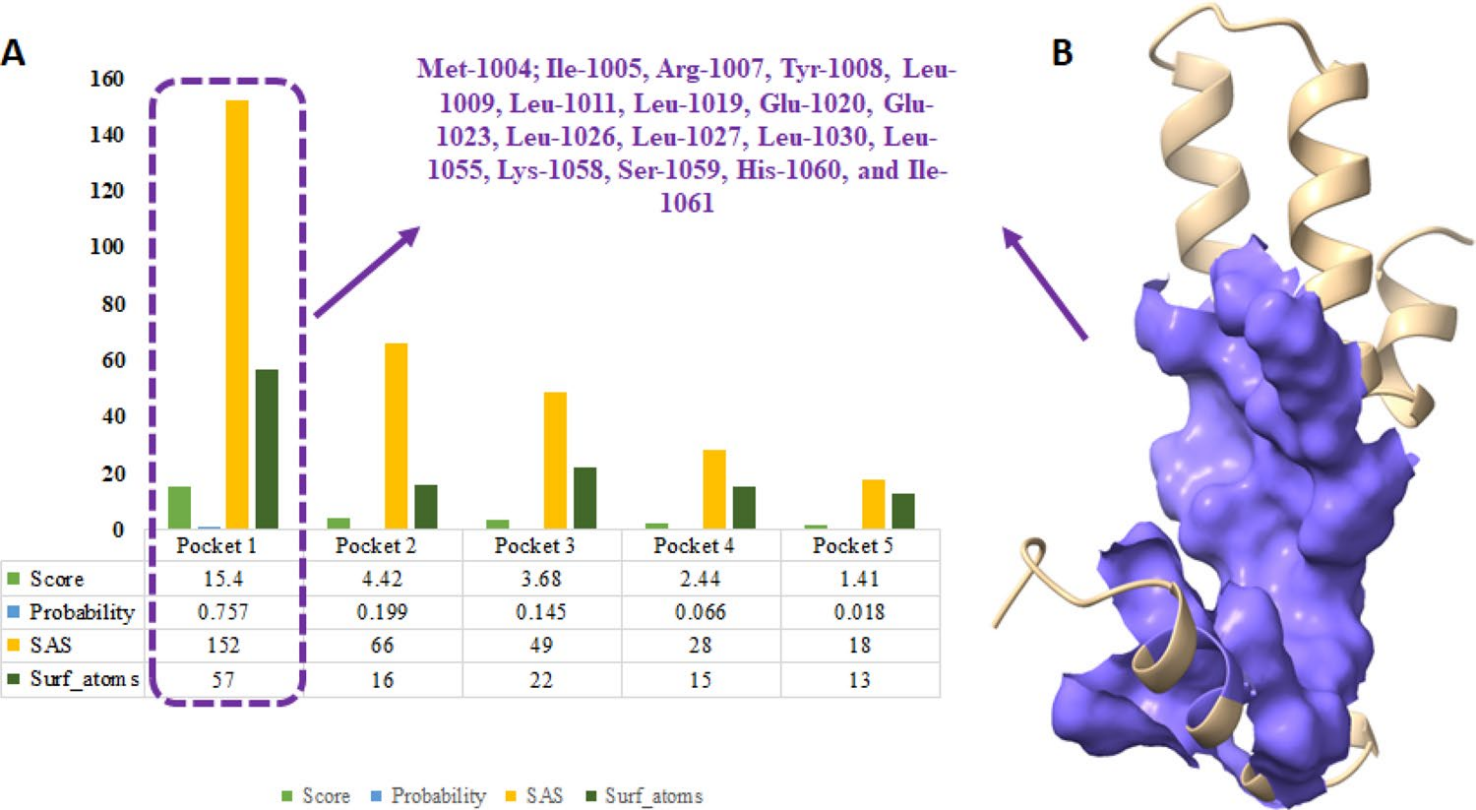
(**A**) Predicted binding pockets represented by graphs lines and scoring values of death domain of MADD protein. (**B**) the predicted model of death domain and core part has been depicted in purple color in surface format.

Generally, binding pocket is the core position of ligands in the active region of protein^40^. Prankweb is a novel machine learning method for prediction of ligand binding sites inside the protein structure^41^. The predicted results showed five different binding pockets with different scoring values 15.4, 4.42, 3.68, 2.44 and 1.41, respectively. The pocket-1 exhibited high score value 15.4 as compared to other binding pockets and constitutes Met-1004, Ile-1005, Arg-1007, Tyr-1008, Leu-1009, Leu-1011, Leu-1019, Glu-1020, Glu-1023, Leu-1026, Leu-1027, Leu-1030, Leu-1055, Lys-1058, Ser-1059, His-1060, and Ile-1061 amino acids. Soluble Accessible Surface (SAS) area represents the area having propensity to interacts with neighboring atoms. The pocket 1 showed good SAS value 152 as compared to other binding pockets values (66, 49, 28 and 18, respectively) with different amino acids of death domain of MADD protein. Moreover, the probability value of pocket 1 was also better as compared to other probabilities values (Fig. 6A, B).

### Shape based screening and retrieval of similar FDA approved drugs

In drug-repositioning approach, shape-based screening, pharmacogenomics and molecular docking simulation are significant parameters to predict the possible drugs from FDA approved medications by considering protein targets^5,42^. Donepezil and galantamine were used as standard drug against AD and used as template to screen FDA approved drug having similar skeleton similarity. In our computational results, Swiss-Similarity results showed 282 drugs were retrieved with donepezil and 351 against galantamine. The scoring values FDA approved drugs based on structural similarity scoring values range from 1.000 to 0.010 and 1.000 to 0.134 for donepezil and galantamine, respectively (Supplementary Tables S2 and S3). The screened drugs were ranked based on similarity scoring values, ranged for 0–1. The 0 value represents dissimilarity between compounds whereas, 1 is used for highly identical compounds in screening approach^5^. In our computational results, drugs depicted good similarity scoring values range from 1 to 0.111. In donepezil screening results, DB07701 and DB3393 were exhibited scoring values 0.999 and 0.837, respectively. Furthermore, in galantamine screening results, it has been observed that DB00318 (0.979), DB01466 (0.977), and DB11490 (0.974) showed scoring values and DB14703 depicted lowest structure similarity result 0.134. The top 100 screened drugs from each standard (donepezil and galantamine) were selected for molecular docking analysis to predict the most suitable candidate having good binding affinity values against MADD protein.

### Virtual screening using PyRx

PyRx is a virtual screening software for computational drug discovery that can be used to screen libraries of compounds against potential drug targets. In PyRx, donepezil-MADD docking results, among 100 screened drugs (Table S4), 72 drugs showed higher docking energy values as compared to standard donepezil (Table 3). Similarly, In PyRx, galantamine-MADD docking results, among 100 screened drugs (Table S5), 67 drugs showed higher docking energy values as compared to standard galantamine (Table 4).

**Table 3:** Screening of FDA approved drugs using donepezil.

Drugbank IDs	Drugs	Binding affinity (Kcal/mol)	Drugbank IDs	Drugs	Binding affinity (Kcal/mol)
DB00496	Darifenacin	− 8.1	DB08927	Amperozide	− 7.7
DB00637	Astemizole	− 8.4	DB08950	Indoramin	− 8.6
DB01199	Tubocurarine	− 8.5	DB09063	Ceritinib	− 8
DB01238	Aripiprazole	− 7.9	DB09083	Ivabradine	− 7.5
DB02929	K201 free base	− 7.7	DB11376	Azaperone	− 7.7
DB04835	Maraviroc	− 7.7	DB11732	Lasmiditan	− 8.8
DB04842	Fluspirilene	− 8.5	DB11793	Niraparib	− 8.1
DB04872	Osanetant	− 9.2	DB12082	Vesnarinone	− 8.2
DB04881	Elacridar	− 10.2	DB12096	PF-05175157	− 8.3
DB05171	E-2012	− 7.9	DB12289	DB12289	− 8.6
DB05414	Pipendoxifene	− 8.4	DB12341	LY-2456302	− 8.3
DB05422	OPC-14523	− 7.5	DB12408	PF-03635659	− 8.4
DB05713	LY-517717	− 9.1	DB12731	Daporinad	− 7.8
DB06144	Sertindole	− 8.1	DB12837	UK-500001	− 9.2
DB06240	Tariquidar	− 10.4	DB12867	Benperidol	− 8.3
DB06306	Onalespib	− 7.9	DB12886	GSK-1521498	− 10.3
DB06401	Bazedoxifene	− 8.4	DB12981	XL-888	− 8.1
DB06446	Dotarizine	− 8.1	DB13080	Roluperidone	− 8
DB06454	Sarizotan	− 9.2	DB13276	Idanpramine	− 8.8
DB06555	Siramesine	− 9.3	DB13310	Ormeloxifene	− 7.8
DB16182	CXD101	− 8.9	DB13393	Emetine	− 7.8
DB15398	Dihydrocapsiate	− 5.7	DB13403	Oxypertine	− 7.9
DB15688	Vazegepant	− 11.1	DB13511	Clebopride	− 7.4
DB14641	Estriol tripropionate	− 7.3	DB13687	Niaprazine	− 7.4
DB08810	Cinitapride	− 7.6	DB13766	Lidoflazine	− 8.8
DB15120	GSK-239512	− 8.3	DB13790	Fipexide	− 7.5
DB15377	ATB-346	− 7.7	DB13865	Dehydroemetine	− 7.6
DB16080	Acolbifene	− 9	DB13954	Estradiol cypionate	− 8
DB16124	Chiauranib	− 9.2	**DB00843**	**Donepezil**	− **7.4**

Significant values are in [bold].

**Table 4:** Screening of FDA approved drugs using galamtamine against MADD.

Drugbank IDs	Drugs	Binding affinity (Kcal/mol)	Drugbank IDs	Drugs	Binding affinity (Kcal/mol)
DB00295	Morphine	− 7.1	DB06444	Dexanabinol	− 6.8
DB00318	Codeine	− 7.4	DB06578	Tonabersat	− 7.5
DB00424	Hyoscyamine	− 7.1	DB07905	Hypothemycin	− 7.6
DB00468	Quinine	− 7.2	DB09039	Eliglustat	− 7
DB00497	Oxycodone	− 7.5	DB09184	Edivoxetine	− 6.7
DB00521	Carteolol	− 6.7	DB09196	Lubazodone	− 6.7
DB00572	Atropine	− 7	DB09209	Pholcodine	− 7.7
DB00611	Butorphanol	− 7.3	DB11181	Homatropine	− 7
DB00654	Latanoprost	− 6.7	DB11411	Fenprostalene	− 6.9
DB00688	Mycophenolate mofetil	− 7.2	DB11490	Nalorphine	− 6.8
DB00704	Naltrexone	− 7.6	DB11711	Navarixin	− 7.7
DB00844	Nalbuphine	− 7.6	DB12057	ORM-12741	− 7.3
DB00905	Bimatoprost	− 7.5	DB12179	Secoisolariciresinol	− 6.6
DB00908	Quinidine	− 6.9	DB12464	Bevenopran	− 7.5
DB00921	Buprenorphine	− 6.8	DB12543	Samidorphan	− 6.8
DB00973	Ezetimibe	− 7.8	DB12637	Onapristone	− 7.5
DB01183	Naloxone	− 7.2	DB12884	Lavoltidine	− 7.4
DB01192	Oxymorphone	− 7.3	DB13471	Nalfurafine	− 8
DB01229	Paclitaxel	− 7.6	DB13718	Hydroquinine	− 7.3
DB01346	Quinidine barbiturate	− 7.2	DB14035	Englitazone	− 8
DB01450	Dihydroetorphine	− 7.4	DB14881	Oliceridine	− 7
DB01466	Ethylmorphine	− 7.1	DB15241	Methylsamidorphan	− 7.3
DB01469	Acetorphine	− 7.2	DB15300	Hydroquinidine	− 7.2
DB01477	Codeine methylbromide	− 7	DB15439	Navoximod	− 7.2
DB01480	Cyprenorphine	− 7	DB15495	Rocaglamide	− 7.2
DB01497	Etorphine	− 7.5	DB15496	Didesmethylrocaglamide	− 7.8
DB01505	Etoxeridine	− 5.7	DB16243	TBA-7371	− 7.7
DB01512	Hydromorphinol	− 6.7	DB16287	Penequinine, Penehyclidine	− 7.2
DB01548	Diprenorphine	− 7.2	DB16351	Volinanserin	− 6.6
DB01551	Dihydrocodeine	− 7.5	DB04865	Omacetaxine mepesuccinate	− 7.4
DB01565	Dihydromorphine	− 6.7	DB04861	Nebivolol	− 7.7
DB01573	Benzylmorphine	− 8.3	DB06217	Vernakalant	− 7
DB02205	Rutamarin alcohol	− 7.1	DB06230	Nalmefene	− 7.3
DB04509	N-Methylnaloxonium	− 7.9	DB00674	**Galantamine**	− **6.7**

Significant values are in [bold].

### Pharmacogenomics analysis

Based on swiss similarity and virtual screening result, the selected drugs further undergo for pharmacogenomics analysis to check their possible interaction with genes encoded proteins and their association with AD. Multiple reports also justify this approach as worthwhile to predict the possible drug having repositioned functionality^5,12^. All the screened drug from both standard (donepezil and galantamine) exhibited good interaction scoring values with predicted genes which showed good correlation with AD. From donepezil screening, our results showed that 10 from 35 drugs exhibited good interaction scoring values and association with AD. The darifenacin showed good interaction scoring values with CHRM3 (0.85), CHRM2 (0.38), CHRM1 (0.29) and CYP2D6 (0.04) which have correlation with AD. The other drug astemizole showed interaction with multiple genes (14) which have association with multiple diseases. The 9 genes such as CYP2J2, HPSE, ABCB1, PPARD, CYP2D6, IDH1, CYP3A4, AR, and TP53 were directly associated with AD. The predicted results showed that astemizole showed highest interaction score 0.90 as compared to other genes. In tubocurarine gene network, 5 genes were interacted and among these four 2 (CHRNA2 and KCNN2) genes were showed interaction with AD. Both CHRNA2 and KCNN2 genes exhibited good interaction scoring values 6.37 and 4.24, respectively. Another important screened drug which exhibited good interaction prediction with AD is aripiprazole through interaction with different genes. The drug-gene association analysis showed that out of 14 genes, eleven (DRD2, HTR1A, ANKK1, SH2B1, HTR2A, CNR1, FAAH, HTR1B, HTR2C, ABCB1, CYP2D6) possessed good interaction scoring values and are directly involves in AD.

Fluspirilene-gene interactions analysis also showed their intimation with AD by targeting multiple genes. The predicted results showed that six gene such as DRD2, HTR1E, XBP1, HTR2A, HTR1A, and PPARD were showed their association with AD with different interaction scoring values. In elacridar-genetic complex, two genes ABCG2 (1.54) and ABCB1 (0.27) were observed having showed their interaction with AD. Another important screened drug was sertindole which showed interactions multiple genes involves in AD and other diseases. A total of 17 genes were observed showed interaction with sertindole and from 17, eight genes (DRD2 (0.15), HTR2A (0.14), HTR2C (0.17), HTR1E (0.1), HTR1A (0.03), HTR1B (0.05), CYP2D6 (0.01), CYP3A4 (0.01)) showed association with AD with different interaction scoring values. Tariquidar interacted with ABCB1 and ABCG2 with scoring values 0.66 and 1.03, respectively. Sarizotan is another screened drug shows interactions with HTR1A (0.91) and DRD2 (0.36) having good interaction score values with AD. Similarly, fipexide interacted with CYP2D6 (0.03) and CYP3A4 (0.02) also depicted good correlation with AD (Table 5).

**Table 5:** Pharmacogenomic analysis of donepezil.

Drugs	Genes	Interaction score	Disease associations	Refs.
Darifenacin	CHRM3	0.85	AD	^43^
	CHRM4	0.54	Schizophrenia	^44^
	CHRM5	0.5	Schizophrenia	^45^
	CHRM2	0.38	AD	^46^
	CHRM1	0.29	AD	^47^
	CYP2D6	0.04	AD	^48^
Astemizole	EED	9.09	Cohen-Gibson	^49^
	HRH1	0.35	Allergic rhinitis	^50^
	CYP2J2	0.91	AD	^51^
	KCNH1	0.76	Epilepsy	^52^
	HPSE	0.7	AD	^53^
	KCNH2	0.15	Short QT syndrome	^54^
	ABCB1	0.02	AD	^55^
	PPARD	0.03	AD	^56^
	CYP2D6	0.01	AD	^57^
	IDH1	0.01	AD	^58^
	GMNN	0.01	Meier-Gorlin syndrome	^59^
	CYP3A4	0.01	AD	^57^
	AR	0.01	AD	^60^
	TP53	0.01	AD	^61^
Tubocurarine	ZACN	12.73	Neoplasm	^62^
	CHRNA2	6.37	AD	^63^
	KCNN2	4.24	AD	^64^
	KCNN3	3.18	Neoplasm metastais	^65^
	KCNN1	2.55	Leukemia	^66^
Aripiprazole	TAAR6	7.96	Bipolar Disorder	^67^
	DRD2	0.83	AD	^68^
	HTR1A	0.38	AD	^69^
	MC4R	1.11	Obesity	^70^
	ANKK1	0.99	AD	^68^
	SH2B1	0.88	AD	^71^
	HTR2A	0.22	AD	^72^
	HTR1D	0.2	Adenocarcinoma	^73^
	CNR1	0.41	AD	^74^
	FAAH	0.38	AD	^75^
	HTR1B	0.18	AD	^76^
	HTR2C	0.12	AD	^72^
	ABCB1	0.03	AD	^55^
	CYP2D6	0.01	AD	^57^
Maraviroc	CCR5	27.58	AD	^77^
Fluspirilene	DRD2	0.13	AD	^68^
	HTR1E	0.09	AD	^72^
	XBP1	0.15	AD	^78^
	HTR1D	0.05	Adenocarcinoma	^73^
	HTR2A	0.04	AD	^72^
	HTR1A	0.03	AD	^69^
	HRH1	0.02	Allergic rhinitis	^50^
	PPARD	0.02	AD	^56^
	THPO	0.04	Amegakaryocytosis	^79^
	NPSR1	0.03	Allergic asthma	^80^
Osanetant	TACR3	3.86	Hypogonadotropic hypogonadism	^81^
	TACR2	3.03	AD	^82^
	TACR1	0.59	Bipolar	^83^
Elacridar	ABCG2	1.54	AD	^84^
	ABCB1	0.27	AD	^55^
LY-517717	F10	5.79	Factor X Deficiency	^85^
Sertindole	DRD2	0.15	AD	^68^
	HTR2A	0.14	AD	^72^
	HTR2C	0.17	AD	^72^
	HTR6	0.31	Schizophrenia	^86^
	KCNH2	0.08	Short QT syndrome	^54^
	ADRA1D	0.21	Crohn Disease	^87^
	HTR1E	0.1	AD	^72^
	HTR1D	0.06	Adenocarcinoma	^73^
	ADRA1B	0.13	Seizures	^88^
	HTR1A	0.03	AD	^69^
	ADRA1A	0.12	Schizophrenia	^89^
	HTR1F	0.12	Schizophrenia	^90^
	HTR1B	0.05	AD	^76^
	HRH1	0.02	Allergic rhinitis	^50^
	DRD4	0.05	Mental Depression	^91^
	CYP2D6	0.01	AD	^48^
	CYP3A4	0.01	AD	^57^
Tariquidar	ABCB1	0.66	AD	^55^
	ABCG2	1.03	AD	^84^
Onalespib	ALK	0.56	Neoplasms	^92^
	HSP90AA1	0.23	Breast Carcinoma	^93^
Bazedoxifene	IL6ST	10.61	Rheumatoid Arthritis	^94^
	IL6R	3.54	Rheumatoid Arthritis	^95^
	ESR2	0.69	Breast neoplasm	^96^
	ESR1	0.32	Breast neoplasm	^96^
Sarizotan	HTR1A	0.91	AD	^69^
	DRD2	0.36	AD	^68^
CXD101	HTT	0.32	Huntington Disease	^97^
Acolbifene	ESR2	0.46	Breast neoplasm	^96^
	ESR1	0.32	Breast neoplasm	^96^
Chiauranib	AURKB	0.27	Liver carcinoma	^98^
	FLT1	0.19	Breast neoplasm	^99^
	FLT4	0.18	Milroy Disease	^100^
	KDR	0.12	Hemangioma	^101^
Amperozide	DRD1	0.19	Bipolar Disorder	^102^
	CYP3A4	0.04	AD	^57^
Indoramin	ADRA1A	0.59	Schizophrenia	^89^
	ADRA1D	0.45	Crohn Disease	^87^
	ADRA1B	0.27	Seizures	^88^
Ceritinib	EML4	4.77	Lung Carcinoma	^100^
	ALK	4.37	Neoplasms	^92^
	TSSK1B	1.99	Systemic Scleroderma	^103^
	NPM1	1.52	Leukemia	^104^
	ROS1	0.73	Adenocarcinoma of lung	^105^
	IGF1R	0.42	Fetal Growth Retardation	^106^
	INSR	0.26	Donohue Syndrome	^107^
	MAP2K1	0.21	Cardio-facio-cutaneous syndrome	^108^
	SRC	0.18	Thrombocytopenia	^109^
	CYP3A4	0.01	AD	^57^
	FLT3	0.06	Leukemia	^110^
	ABCB1	0.04	AD	^55^
Ivabradine	HCN3	4.24	Diabetes Mellitus	^111^
	MALAT1	4.24	Breast neoplasm	^112^
	HCN1	3.18	Epileptic encephalopathy	^113^
	HCN4	2.55	Sick Sinus Syndrome	^114^
	CYP3A4	0.01	AD	^57^
Lasmiditan	HTR1F	10.97	Schizophrenia	^90^
Niraparib	PARP2	6.06	Lipodystrophy	^115^
	SLFN11	4.71	Malignant Neoplasms	^116^
	PARP1	3.54	Breast neoplasm	^117^
	BRCA1/2	1.41	Breast neoplasm	^118^
	ATR	0.79	Seckel syndrome	^119^
	ATM	0.13	Ataxia Telangiectasia	^120^
	PTEN	0.07	Hamartoma Syndrome	^121^
	IDH1	0.04	AD	^58^
Vesnarinone	THBS1	2.27	Diabetic Retinopathy	^122^
	NT5E	1.3	Calcification of Joints	^123^
	FAS	0.96	Autoimmune Lymphoproliferative Syndrome	^124^
	PDE3A	0.4	Brachydactyly	^125^
	CYP3A4	0.02	AD	^57^
	KCNH2	0.03	Short QT syndrome	^54^
	TP53	0.05	AD	^61^
PF-05175157	ACACB	21.22	Obesity	^126^
LY-2456302	OPRM1	0.17	Alcoholic Intoxication	^127^
	OPRD1	0.33	Alcoholic Intoxication	^127^
Daporinad	NAMPT	42.43	Colorectal Carcinoma	^128^
Benperidol	DRD4	0.33	Mental Depression	^91^
	CYP2D6	0.04	AD	^48^
	DRD2	0.12	AD	^68^
GSK-1521498	OPRM1	0.51	Alcoholic Intoxication	^127^
XL-888	NRAS	0.66	Colorectal Carcinoma	^129^
	HSP90AB1	0.42	Pulmonary Fibrosis	^130^
	BRAF	0.33	Melanoma	^94^
Emetine	HIF1A	0.19	Hypertensive disease	^131^
	MTOR	0.1	Focal cortical dysplasia	^132^
	ATXN2	0.05	Spinocerebellar Ataxia-II	^133^
	ATAD5	0.04	Carcinoma, Ovarian Epithelial	^134^
	AR	0.01	Malignant neoplasm of prostate	^135^
Clebopride	CYP2D6	0.11	AD	^48^
Lidoflazine	SLC29A1	1.14	Colorectal Carcinoma	^136^
	KCNH2	0.19	Short QT syndrome	^54^
	SCN3A	0.27	Epilepsies	^137^
	CYP2D6	0.03	AD	^48^
Fipexide	CYP2D6	0.03	AD	^48^
	CYP3A4	0.02	AD	^57^
	CYP1A2	0.03	Liver carcinoma	^138^
	CYP2C19	0.03	Depressive disorder	^139^
Estradiol cypionate	ESR1	0.16	Breast neoplasm	^96^
	AR	0.04	Malignant neoplasm	^135^

In galantamine pharmacogenomics results, forty screened drugs having good similarity score has been predicted having possible correlation with AD mediated genes. However, from forty, eight screened drugs have been elected having good interaction scoring values as well as exhibited good association with AD mediated genes. Morphine shows interactions with 24 genes having association with different diseases and only seven genes possessed association with AD. These seven genes PDYN (4.11), OPRK1 (0.23), PER1 (2.05), HMOX2 (2.05), ABCB1 (0.09), CYP2D6 (0.01), DRD2 (0.02) may predict the good therapeutic behavior of morphine as an anti-AD drug. Codeine is another screened drug extracted through galantamine that could be use as repositioned drug against AD. Our drug-gene predicted results showed that eight genes OPRD1 (1.22), OPRM1 (0.58), OPRK1 (0.63), UGT2B7 (0.76), ABCB1 (0.13), CYP3A4 (0.02), CYP2D6 (0.01), and AR (0.01) were interacted having different interaction values. The literature mining showed that four genes among eight were involved in AD. Quinine-gene interaction analysis showed that CYP3A7 (1.27), SLC29A4 (0.64), COP1 (1.27), IL2 (0.6), G6PD (0.18), ABCB1 (0.03), CYP3A4 (0.01), and CYP2D6 (0.01). Moreover, from gene literature mining it has been observed that 5 genes were involved in AD. Similarly, atropine, nalbuphine, quinidine, volinanserin and vernakalant are also screened drugs interacting with different genes which are linked with AD (Table 6).

**Table 6:** Pharmacogenomic analysis of galantamine.

Drugs	Genes	Interaction score	Disease associations	Refs.
Morphine	PDYN	4.11	AD	^140^
	SYP	4.11	Mental retardation	^141^
	OPRD1	0.32	Alcoholic Intoxication	^127^
	COMT	1.09	Bipolar Disorder	^142^
	UGT2B7	0.78	Malignant neoplasm	^143^
	RHBDF2	1.37	Esophageal cancer	^144^
	OPRM1	0.2	Alcoholic Intoxication	^127^
	OPRK1	0.23	AD	^145^
	TAOK3	2.05	Neoplasm Metastasis	^146^
	PER1	2.05	AD	^147^
	HMOX2	2.05	AD	^148^
	LPAR2	1.37	Breast neoplasm	^149^
	KCNJ6	0.82	Alcoholic Intoxication, Chronic	^150^
	GRM1	0.82	Schizophrenia	^151^
	ABCB1	0.09	AD	^55^
	F2R	0.26	Stomach neoplasm	^152^
	GCG	0.26	Diabetes Mellitus	^153^
	ITGAM	0.41	Lupus Erythematosus	^154^
	NR4A1	0.41	Pancreatic neoplasm	^155^
	CCR2	0.37	Pulmonary Fibrosis	^156^
	SLC22A1	0.27	Liver carcinoma	^157^
	CCKBR	0.2	Panic Disorder	^158^
	CYP2D6	0.01	AD	^57^
	DRD2	0.02	AD	^68^
Codeine	OPRD1	1.22	Alcoholic Intoxication	^127^
	OPRM1	0.58	Alcoholic Intoxication	^127^
	OPRK1	0.63	AD	^145^
	UGT2B7	0.76	Neoplasm urinary bladder	^143^
	ABCB1	0.13	AD	^55^
	CYP3A4	0.02	AD	^57^
	CYP2D6	0.01	AD	^57^
	AR	0.01	Prostate neoplasm	^135^
Hyoscyamine	CHRM5	0.99	Schizophrenia	^45^
Quinine	CYP3A7	1.27	Malignant Neoplasms	^159^
	SLC29A4	0.64	AD	^160^
	COP1	1.27	Neoplasms	^161^
	IL2	0.6	AD	^162^
	G6PD	0.18	Anemia	^163^
	ABCB1	0.03	AD	^55^
	CYP3A4	0.01	AD	^57^
	CYP2D6	0.01	AD	^57^
Oxycodone	RHBDF2	4.71	Esophageal cancer	^144^
	COMT	2.87	Bipolar Disorder	^142^
	OPRD1	0.65	Alcoholic Intoxication	^127^
	OPRM1	0.57	Alcoholic Intoxication	^127^
	OPRK1	0.56	AD	^145^
	UGT2B7	0.67	Neoplasm of urinary bladder	^143^
	NR1I3	1.01	Drug-Induced Liver Disease	^164^
	CYP3A5	0.21	Malignant neoplasm of prostate	^165^
	ABCB1	0.09	AD	^55^
Carteolol	ADRB1	3.11	Hypertensive disease	^166^
	ADRB2	1.61	Asthma	^167^
Atropine	GUCA2A	3.54	Carcinogenesis	^168^
	CHRM5	0.66	Schizophrenia	^45^
	F2R	0.66	Stomach neoplasm	^152^
	CHRM2	0.64	AD	^46^
	CHRM4	0.54	Schizophrenia	^44^
	CHRM1	0.43	AD	^47^
	CYP3A5	0.1	Prostate neoplasm	^165^
	CHRM3	0.37	AD	^43^
	AR	0.01	Prostate neoplasm	^135^
Butorphanol	OPRD1	1.54	Alcoholic Intoxication	^127^
	OPRK1	1.33	AD	^145^
	OPRM1	0.88	Alcoholic Intoxication	^127^
	CYP3A4	0.02	AD	^57^
	CYP1A2	0.03	Liver carcinoma	^138^
	CYP2C9	0.04	Unipolar Depression	^169^
	CYP2C19	0.04	Depressive disorder	^139^
Latanoprost	PTGFR	19.97	Breast Carcinoma	^170^
	ABCC4	1.46	Malignant neoplasm of prostate	^171^
	PTGS1	0.64	Hyperalgesia	^172^
Mycophenolate mofetil	IMPDH2	1.18	Neoplasm Metastasis	^173^
	ITGB2	1.12	Leukocyte adhesion deficiency type 1	^174^
	IMPDH1	1.06	Neoplasm Metastasis	^173^
	CSF2	0.78	Rheumatoid Arthritis	^175^
	AR	0.01	Prostate neoplasm	^135^
Naltrexone	OPRD1	1.47	Alcoholic Intoxication	^127^
	OPRM1	1.35	Alcoholic Intoxication	^127^
	OPRK1	0.8	AD	^145^
	GCG	0.99	Diabetes Mellitus	^153^
	DBH	1.14	dopamine beta hydroxylase deficiency	^176^
	BAX	0.33	AD	^177^
Nalbuphine	OPRK1	1.79	AD	^145^
	OPRM1	1.11	Alcoholic Intoxication	^127^
	OPRD1	0.98	Alcoholic Intoxication	^127^
	CYP2D6	0.04	AD	^48^
	CYP3A4	0.02	AD	^57^
	CYP1A2	0.04	Liver carcinoma	^138^
Bimatoprost	PTGFR	14.98	Breast Carcinoma	^170^
	PTGER3	5.3	Stevens-Johnson Syndrome	^178^
	PTGER1	3.54	Breast Carcinoma	^170^
Quinidine	KCNK5	1.68	Balkan Nephropathy	^179^
	KCNU1	1.68	Diabetes Mellitus	^180^
	CYP3A7	0.67	Malignant Neoplasms	^159^
	KCNT1	0.84	Epileptic encephalopathy	^181^
	KCNK16	0.84	Diabetes Mellitus	^182^
	SLC29A4	0.34	AD	^160^
	KCNH5	0.67	Epilepsy	^183^
	KCNE1	0.67	Long qt syndrome 5	^184^
	KCNA7	0.42	Familial heart attack	^185^
	SCN5A	0.41	Brugada Syndrome	^186^
	KCNH1	0.28	Temple-Baraitser Syndrome	^187^
	ABCB1	0.04	AD	^55^
	CYP3A4	0.02	AD	^57^
	CYP2D6	0.01	AD	^48^
	CYP2C9	0.02	Unipolar Depression	^169^
	CYP1A2	0.01	Liver carcinoma	^138^
	CYP2C19	0.01	Depressive disorder	^139^
Buprenorphine	OPRD1	0.88	Alcoholic Intoxication	^127^
	OPRM1	0.62	Alcoholic Intoxication	^127^
	OPRK1	0.43	AD	^145^
	UGT2B7	0.61	Neoplasm of urinary bladder	^143^
	COMT	0.4	Bipolar Disorder	^142^
	CYP3A5	0.12	Malignant neoplasm of prostate	^165^
	CYP3A4	0.02	AD	^57^
Ezetimibe	NPC1L1	175.04	Diabetes Mellitus	^188^
	NPSR1	0.38	Asthma	^189^
Naloxone	OPRD1	1.07	Alcoholic Intoxication	^127^
	OPRM1	0.89	Alcoholic Intoxication	^127^
	OPRK1	0.33	AD	^145^
	GRP	1.65	Head Neoplasms	^190^
	NTF3	0.72	Schizophrenia	^191^
	RARA	0.37	Promyelocytic Leukemia	^192^
	BDNF	0.18	Congenital hypoventilation	^193^
Oxymorphone	OPRM1	1.03	Alcoholic Intoxication	^127^
	OPRD1	0.73	Alcoholic Intoxication	^127^
	NR1I3	2.27	Drug-Induced Liver Disease	^164^
	CYP2D6	0.03	AD	^48^
Paclitaxel	STMN1	1.31	Liver carcinoma	^194^
	SPATA5	1.31	Epilepsy	^195^
Ethylmorphine	CYP2D6	0.11	AD	^48^
Etorphine	OPRD1	2.61	Alcoholic Intoxication	^127^
	OPRM1	1.2	Alcoholic Intoxication	^127^
	OPRK1	0.95	AD	^145^
Diprenorphine	OPRD1	2.28	Alcoholic Intoxication	^127^
	OPRK1	0.48	AD	^145^
	OPRM1	0.34	Alcoholic Intoxication	^127^
Dihydromorphine	OPRD1	1.31	Alcoholic Intoxication	^127^
	OPRK1	0.95	AD	^145^
	OPRM1	0.68	Alcoholic Intoxication	^127^
Dexanabinol	GLRA2	1.74	Autistic Disorder	^196^
	GLRA3	0.83	Autistic Disorder	^196^
	GLRA1	0.83	Autistic Disorder	^196^
	CNR1	0.15	AD	^74^
Tonabersat	HTR1D	2.12	Adenocarcinoma	^73^
Eliglustat Edivoxetine	UGCG	15.91	Liver Cirrhosis	^197^
	CYP2D6	0.16	AD	^48^
Nalorphine	OPRD1	0.33	Alcoholic Intoxication	^127^
	OPRK1	0.24	AD	^145^
ORM-12741	OPRM1	0.17	Alcoholic Intoxication	^127^
	ADRA2C	0.77	Heart failure	^198^
Bevenopran	OPRM1	1.03	Alcoholic Intoxication	^127^
Samidorphan	OPRM1	0.34	Alcoholic Intoxication	^127^
	OPRD1	0.33	Alcoholic Intoxication	^127^
	OPRK1	0.24	AD	^145^
Onapristone	PGR	1.14	Endometriosis	^199^
	NR3C2	0.92	Pseudohypoaldosteronism	^200^
Nalfurafine	NR3C1	0.32	Pseudohypoaldosteronism	^200^
	OPRK1	0.72	AD	^145^
Oliceridine	OPRM1	0.51	Alcoholic Intoxication	^127^
Methylsamidorphan	OPRM1	0.51	Alcoholic Intoxication	^127^
Navoximod	TDO2	7.07	Schizophrenia	^201^
Rocagla	NFE2L2	0.08	Lung Carcinoma	^202^
Volinanserin	HTR2A	0.44	AD	^72^
	HTR2B	0.31	AD	^72^
	HTR2C	0.22	AD	^72^
Nebivolol	ADRB1	1.21	Hypertensive disease	^166^
	ADRB2	0.23	Asthma	^167^
	CYP2D6	0.05	AD	^48^
Vernakalant	CYP2D6	0.11	AD	^48^
Nalmefene	OPRD1	1.31	Alcoholic Intoxication	^127^
	OPRK1	0.95	AD	^145^
	OPRM1	0.34	Alcoholic Intoxication	^127^

### Molecular docking using autodock

#### Docking energy analysis against death domain of MADD

The selected drugs-death domain docked complexes were analyzed based on lowest binding energy values (Kcal/mol) and hydrogen/hydrophobic interaction analyses. Results showed that all drugs showed good binding energy values (Kcal/mol) and binds within the active site with appropriate conformational poses (Table 7). Docking energy values is most significant parameter to screen and evaluate the drugs in binding with target proteins^5,203–206^. Among all, six drugs including darifenacin (− 7.59 kcal/mol), astemizole (− 7.19), tubocurarine (− 8.26), elacridar (− 7.72), sertindole (− 7.58) and tariquidar (− 8.42) showed higher than − 7 (kcal/mol) docking energy values. The ligand efficiency is a useful metric for lead selection using computational resources^207^. Ligand efficiency is a way of normalizing the potency and MW of a compound to provide a useful comparison between compounds with a range of MWs and activities. In our docking results, the predicted ligand efficiency (LE) results, showed that all drugs exhibited good LE values, whereas lowest value is considered as most significant compared to other. The comparative results showed that Tubocurarine, Elacridar and Tariquidar exhibited − 0.18 value which was lowest compared to rest of all drugs.

**Table 7:** The predicted docking energy values of selected drugs.

Docking complexes	Docking energy (Kcal/mol)	Ligand efficiency (∆g)	Inhibition constant (uM)	Intermol energy (Kcal/mol)	Total internal (Kcal/mol)	Torsional energy (Kcal/mol)
Morphine	− 6.31	− 0.30	23.72	− 6.91	− 0.39	0.6
Codeine	− 6.60	− 0.30	14.55	− 7.2	− 0.78	0.6
Quinine	− 6.21	− 0.26	28.07	− 7.7	− 1.1	1.49
Darifenacin	− 7.59	− 0.24	2.73	− 9.68	− 2.5	2.09
Atropine	− 5.41	− 0.26	107.92	− 7.2	− 1.12	1.79
Astemizole	− 7.19	− 0.21	5.37	− 9.58	− 1.7	2.39
Nalbuphine	− 6.36	− 0.24	21.81	− 7.85	− 2.35	1.49
Quinidine	− 6.47	− 0.27	17.94	− 7.97	− 1.19	1.49
Tubocurarine	− 8.26	− 0.18	888.7	− 9.45	− 0.86	1.19
Aripiprazole	− 6.48	− 0.22	17.76	− 8.57	− 1.62	2.09
Fluspirilene	− 6.91	− 0.20	8.57	− 9.0	− 2.29	2.09
Elacridar	− 7.72	− 0.18	2.21	− 10.1	− 1.3	2.39
Sertindole	− 7.58	− 0.24	2.76	− 9.08	− 1.3	1.49
Vernakalant	− 5.59	− 0.22	79.57	− 7.98	− 1.96	2.39
Tariquidar	− 8.42	− 0.18	671.0	− 11.7	− 2.42	− 2.42
Sarizotan	− 6.86	− 0.26	9.35	− 8.35	− 2.25	1.49
Fipexide	− 6.35	− 0.26	22.11	− 8.14	− 1.05	− 1.05
Volinanserin	− 5.80	− 0.21	56.06	− 8.19	− 1.69	− 1.69

Another significant parameter in drug analysis is inhibition constant (Ki). Autodock uses the binding energy (Kcal/mol) to calculate the inhibition constant. The binding energy is the free energy change for the protein-inhibitor interaction (ΔG). This is used to determine the inhibition constant (ki) which is, in turn, the dissociation constant (Kd) of the protein-inhibitor complex. In our predicted results, morphine, codeine, quinine, darifenacin, astemizole, nalbuphine, quinidine, aripiprazole, fluspirilene, elacridar, sertindole, vernakalant, sarizotan, fipexide and volinanserin exhibited good inhibition constant value on comparison with standard value 80.08 µM^208^. The intermolecular energy is the attractive intermolecular forces between particles that tend to draw the particles together and evaluates based on minimum energy values. According to AutoDock, the binding energy is the sum of the intermolecular forces acting upon the receptor-ligand complex^209^.

1 $$\Delta {\text{Gbinding }} = \, \Delta {\text{Gvdw }} + \, \Delta {\text{Gelec }} + \, \Delta {\text{GHbond }} + \, \Delta {\text{Gdesolv }} + \, \Delta {\text{Gtorsional}}$$

Here, ΔG gauss: attractive term for dispersion of two gaussian functions, ΔGrepulsion: square of the distance if closer than a threshold value, ΔGhbond: ramp function also used for interactions with metal ions, ΔGhydrophobic: ramp function, ΔGtors: proportional to the number of rotatable bonds.

### Binding pocket and binding interaction analysis

**Figure 7:**
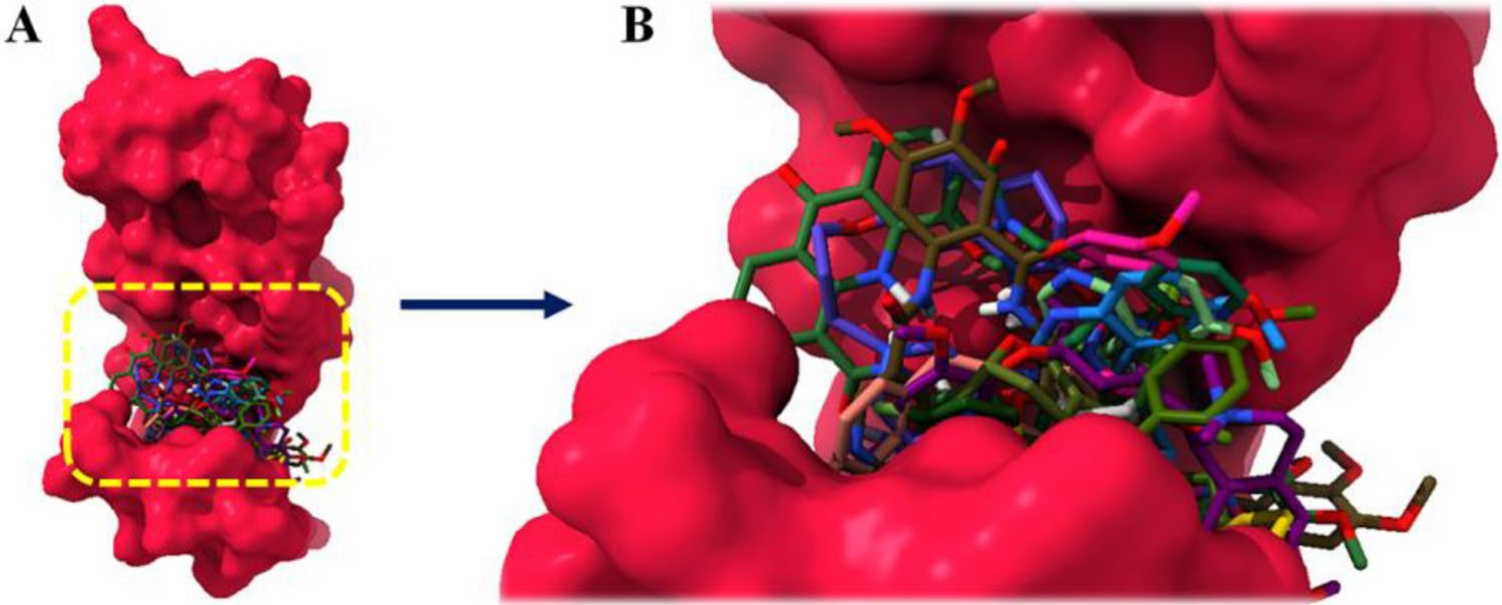
(**A**) Binding pocket of death domain of MADD protein along with all selected drugs. (**B**) In detail description, binding of morphine, codeine, quinine, darifenacin, astemizole, nalbuphine, quinidine, aripiprazole, fluspirilene, elacridar, sertindole, vernakalant, sarizotan, fipexide and volinanserin at the active site of target protein.

The active binding site is a cavity on the surface or in the interior of a protein that possesses suitable properties for ligand binding^40^. 18 drugs were docked against death domain of MADD and superimposed all docking complexes to check the binding conformations inside the binding pocket. The predicted results showed that all 18 drugs were firmly bind within the active region of MADD however, the binding behavior was deviant with each other’s (Fig. 7A, B).

Drug-protein binding interaction is significant parameter to better understand the molecular docking results and to deeply understand the conformational behavior^210,211^. Top six drug having docking energy greater than − 7.00 kcal/mol were selected to check the binding conformation behavior and draggability behavior of MADD. The best six drug such as darifenacin (− 7.59), astemizole (− 7.19), tubocurarine (− 8.26), elacridar (− 7.72), sertindole (− 7.58) and tariquidar (− 8.42) exhibited good docking energy values (Kcal/mol) and binding interaction profiles. In darifenacin docking, single hydrogen bond was observed with appropriate binding distance. The amino group (NH_2_) of darifenacin formed a hydrogen bond with Glu1020 having bond length 2.60 Å within the active region of death domain of MADD structure. Similarly, elacridar and tariquidar also formed hydrogen bonds with Glu1023 having appropriate bond lengths. The nitrogen atom of NH_2_ elacridar formed hydrogen bond with Glu1023 having bond length 3.00 Å, whereas tariquidar formed two hydrogen bonds with Glu1023 having bond distances 2.50 and 2.80 Å, respectively. Glutamic acid is an α-amino acid that is used by almost all living beings in the biosynthesis of proteins. Our docking results showed that screened drugs bind with Glu1020 and Glu1023 which may depict their significance in death domain formation and their involvement in the associated signaling pathways. The reported data showed that hydrogen bond between 2.5 and 3.0 Å may considered as standard bond length in ligand docking which strengthen the docked complexes^212^.

**Figure 8:**
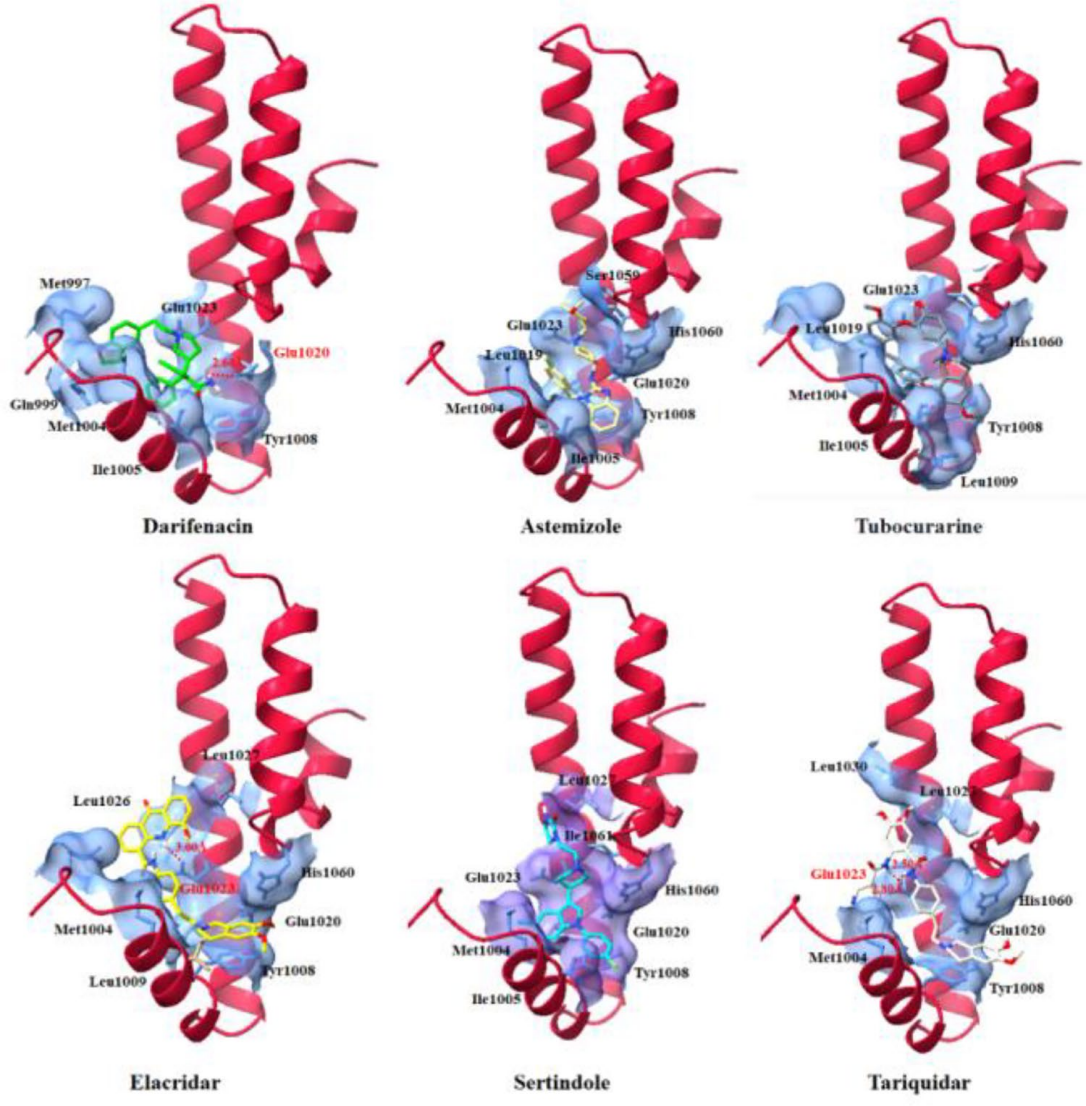
Binding interaction of best darifenacin, astemizole, tubocurarine, elacridar, sertindole and tariquidar death domain of MADD protein along with all selected drug binding.

Therefore, intermolecular hydrogen bonds have good impact in the formation of drug-protein docking complex and enhance their accuracy. In all our docking analysis, darifenacin, elacridar and tariquidar exhibited comparative hydrogen bond length against the MADD protein. The comparative analysis showed that all drugs, darifenacin, astemizole, tubocurarine, elacridar, sertindole and tariquidar were bind at same position inside the active region of death domain in MADD protein. The common interactions pattern among unique residues also showed the reliability of our docking results. Furthermore, in detail results, darifenacin is surrounded by Glu1020, Tyr1008, Ile1005, Met1004, Gln999, Met997 and Glu1023 residues, whereas elacridar and tariquidar also encompassed similar amino acids such as Leu1027, Leu1030, Met1004, Leu1009, Tyr1008, Glu1020, His1060, and Glu1023, respectively. The other three drugs also showed their binding potential at the same binding regions with different conformational symmetry. The occurrence of common amino acids in all docking complexes also ensure the significance of these amino acid particularly Glu1023 is essential for target binding (Fig. 8).

### Molecular dynamic simulation

The best docking complexes were analyzed further through the evaluation of residual flexibility in the target protein by employing MD simulation experiment. The MD simulation study was employed at 100 ns by using Gromacs 5.1.2 by generating root mean square deviations & fluctuations (RMSD/F), radius of gyration (Rg) and solvent accessible surface area (SASA) graphs.

### Root mean square deviation and fluctuation analysis

The protein backbone behavior in the simulation running time were evaluated through RMSD/F graphs. The RMSD graph results of six docking complexes (darifenacin, astemizole, tubocurarine, elacridar, sertindole and tariquidar) showed the steady and little fluctuated behavior through-out the simulation time. The RMSD graph lines displayed an increasing trend with RMSD values ranging from 0 to 0.1 nm from 0 to 100 ns.

Initially, all the graph lines (red, indigo, brown, green, blue, and yellow) of docked complexes showed an increasing trend with RMSD value 0–0.1 nm from 0 to 10 ns. However, in the same simulation time tariquidar graph line showed much stable behavior as compared to other complexes. From 10 to 20 ns, again fluctuated graphs lines were seen whereas, tariquidar (yellow) remained steady and stable at RMSD value around 0.6 nm. Tubocurarine (brown) displayed highest RMSD value (> 1 nm) at 20 ns as compared to all other graphs lines. Sertindole (blue) line showed upward movement from 0 to 10 ns whereas, from 10 to 20 ns the graph line showed downward movement. However, darifenacin and tubocurarine has been exposed in continuously increasing trend with increased RMSD values (nm). Elacridar (green) and astemizole also showed fluctuated behavior at this simulation time frame. From 20 to 40 ns much stable behavior has been observed in the tariquidar (yellow) and elacridar (green) graphs lines, respectively compared to other graph lines. Darifenacin (red), astemizole (indigo), tubocurarine (brown), elacridar (green), and sertindole (blue) showed high fluctuations in graph lines along with deviated RMSD values.

**Figure 9:**
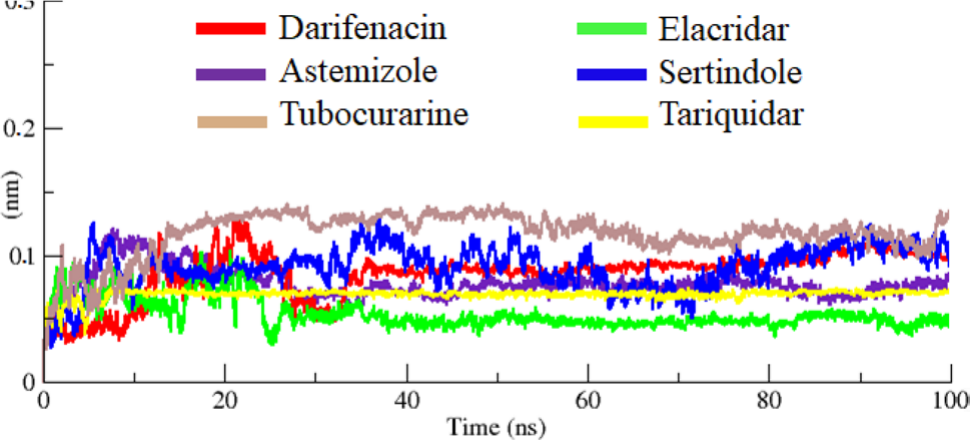
RMSD graph of docked complexes of darifenacin, astemizole, tubocurarine, elacridar, sertindole and tariquidar, respectively from 0 to 100 ns.

From 40 to 100 ns, again tariquidar (yellow) remained displayed stable behavior and no fluctuations has been observed in the backbone of MADD protein in the docking complex. Similarly, elacridar (green) also represented similar results with tariquidar and still remined stable behavior in the docking complex. The rest of all other docking complexes showed little fluctuations with steady stable behavior in the simulation time. The RMSD is used to measure the difference between the backbones of a protein from its initial structural conformation to its final position^213^ and multiple research data exposed different RMSD values range from 0 to 0.3 nm in their MD simulation analysis^203^. The overall RMSD graphs lines showed that all docked complexes showed fluctuated behavior in the simulation time frame. The generated graphs results showed the stable behavior in the backbone of all protein complexes (Fig. 9).

**Figure 10:**
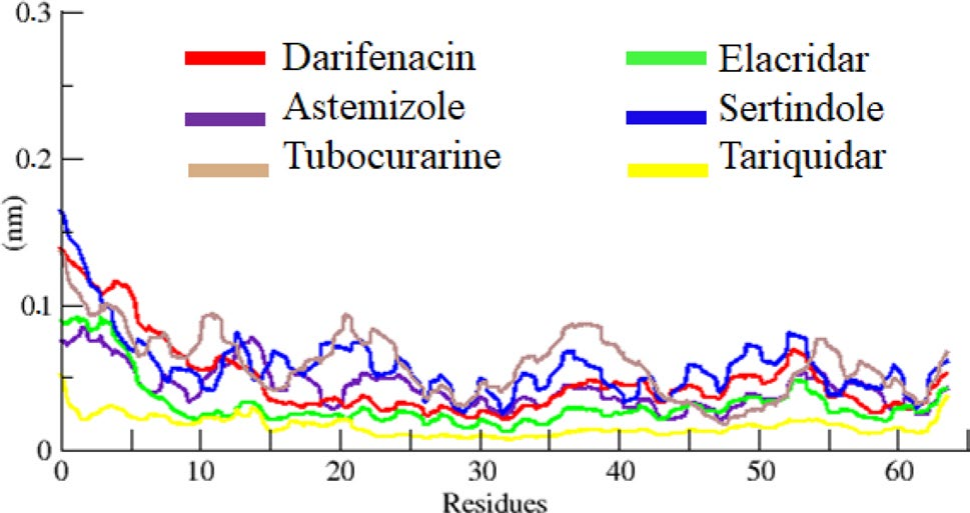
RMSF graph of darifenacin, astemizole, tubocurarine, elacridar, sertindole and tariquidar docking complexes at 100 ns.

The RMSF results of all docked complexes dynamically fluctuated from residues N to C terminals. The protein structures are composed of with different structural architecture. There are different small fluctuations through-out the simulation time frame. However, most of protein complexes remain little stable in the RMSF graph. The tariquidar graph line displayed less fluctuations as compared to all other protein complexes which ensure his stable behavior in the docking complex. The comparative results showed that tubocurarine and sertindole were exhibited higher fluctuations peaks, however, the values remain at 0.1 nm in the simulation study. All the results have been displayed in Fig. 10.

### Radius of gyration and solvent accessible surface area

**Figure 11:**
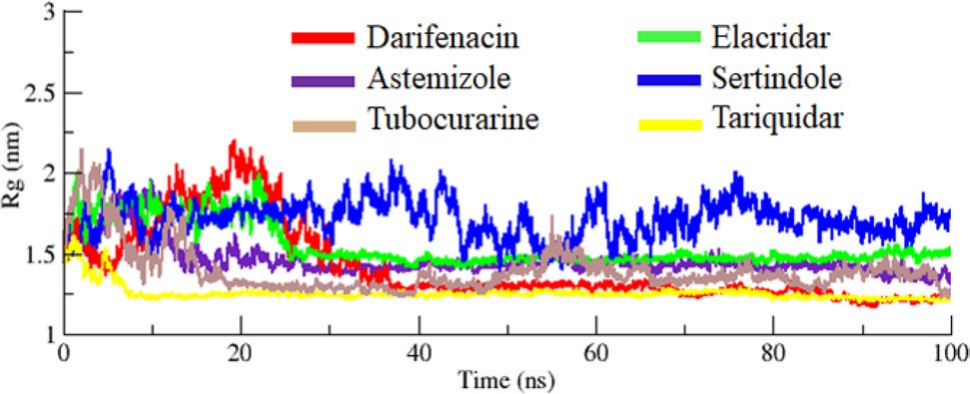
Rg graph of darifenacin, astemizole, tubocurarine, elacridar, sertindole and tariquidar docking complexes from simulation time 0–100 ns.

**Figure 12:**
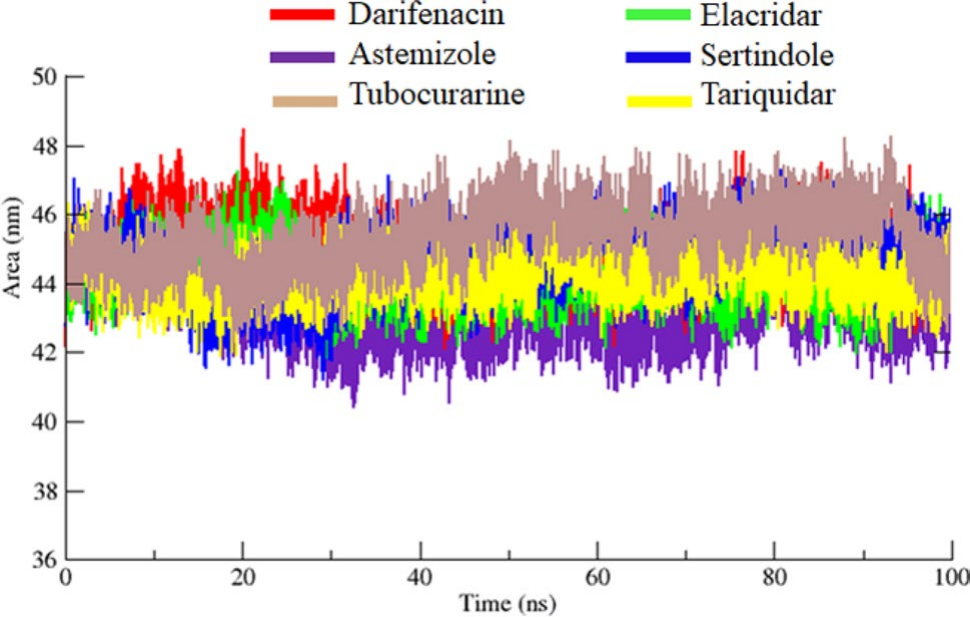
SASA graph of darifenacin, astemizole, tubocurarine, elacridar, sertindole and tariquidar docking structures from 0 to 100 ns simulation time frame.

The structural compactness of protein was calculated by Rg. The generated results depicted that Rg values of all the docked structures showed little variations from 1.25 to 2.25 nm. Initially, the graph lines were unstable and showed little fluctuations from 0 to 20 ns. The comparative analysis showed that sertindole graph line displayed fluctuations from 0 to 100 ns. However, darifenacin, astemizole, tubocurarine, elacridar, and tariquidar graph lines were remined stable with less fluctuations after 20–100 ns. The overall stable behavior was observed at the Rg value 1.5 nm (Fig. 11). The solvent-accessible surface areas (SASA) were also observed and shown in. Results showed that the values of SASA of all five docked complexes were centered on 45 nm^2^ in the simulation time 0–100 ns (Fig. 12).

### Protein–ligand interaction energy

**Figure 13:**
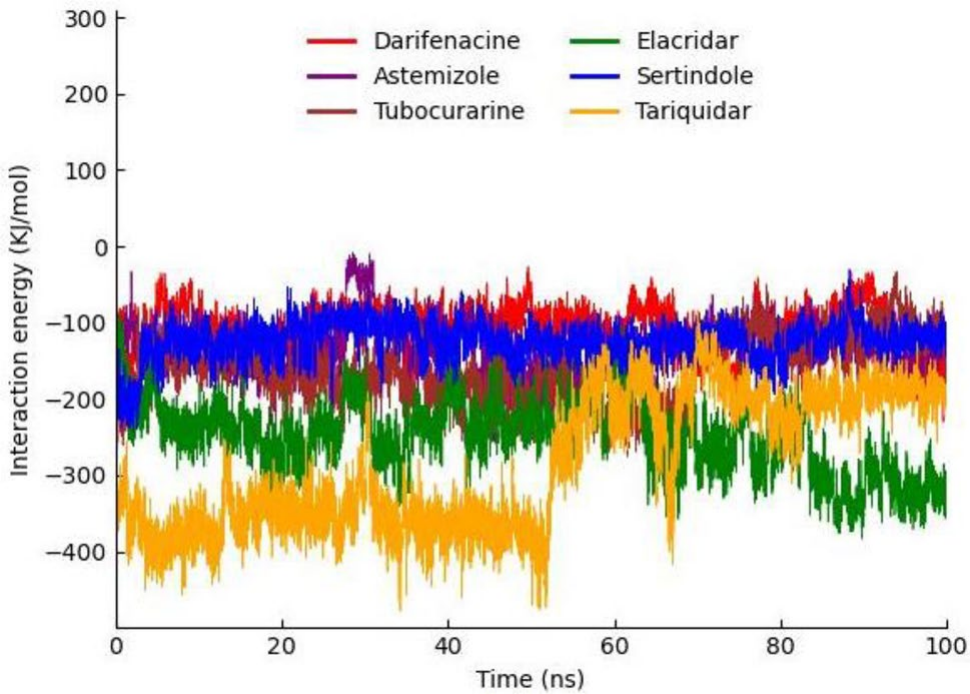
Graphical representation of MD protein–ligand Interaction energy trajectories darifenacin, astemizole, tubocurarine, elacridar, sertindole and tariquidar.

The interaction energy has been calculated from MD trajectories in couple of forms: electrostatic (coulombic) interaction energy and Lennard–Jones interaction energy, with their sum representing the total interaction energy. According to interaction energy analysis tariqudar drug showed the lowest interaction energy − 285.728 followed by elacridar and tobucorarine (− 248.6337 and − 160.0039 respectively). Moreover, the astemizole, sertindole, and darifenacin exhibit high interaction energy as compared to the top (Table 8). Furthermore, the graphical depiction of interaction energy of those six drugs has been carried out to see the trajectory changes of all six drugs throughout 100 ns MD simulation (Fig. 13).

**Table 8:** MD protein–ligand Interaction energy table of all six compounds.

Drugs	Interaction energy		Total interaction energy
	Coulombic	Lennard–Jones	
Astemizole	− 18.1286	− 116.472	− 134.6006‬
Darifenacin	− 10.208	− 93.7889	− 103.9969
Elacridar	− 59.3197	− 189.314	− 248.6337‬
Sertindole	− 11.7399	− 112.683	− 124.4229‬
Tariqudar	− 101.295	− 184.433	− 285.728
Tobucorarine	− 24.7449	− 135.259	− 160.0039

### Binding poses validation

To analyze the interaction of darifenacin, astemizole, tubocurarine, elacridar, sertindole and tariquidar during 100 ns MD simulation; MD binding Pose validation has been carried out against best selected drugs. Snapshots of all simulated drugs results demonstrated that all drugs remained in the active region of MADD during the 100 ns MD simulation and maintained strong interactions with binding pocket residues. The elacridar manifest the strongest interaction while tubocurarine and sertindole also exhibit single hydrogen bonds and in addition to hydrophobic interactions. Furthermore, astemizole, darifenacin, and tariquidar also exhibits strong hydrophobic interactions. The results demonstrate that the drugs remain bounded to the active pocket of the target protein till 100 ns and block the active site residues (Supplementary Fig. S6).

## Conclusion

Novel drug development is time consuming process with relatively high debilitating cost. In present time, drug repositioning is an in-silico approach being employing for drug discovery. In the present research repositioning profiles of known drugs against AD has been explored using shape-based screening, molecular docking pharmacogenomics and MD simulation approaches. Swiss-Similarity results showed 282 drugs were retrieved with donepezil and 351 against galantamine and further evaluated based on similarity scoring values, docking energy values and pharmacogenomics analysis. The detailed pharmacogenomics and extensive data mining showed that three drugs have direct association with AD by targeting different genes. The detailed screening results, showed that darifenacin, astemizole, tubocurarine, elacridar, sertindole and tariquidar drugs exhibited good lead like behavior against AD. Moreover, in MD simulation result tariquidar displayed better stability behavior as compared to rest of other docking complexes drugs with respect to their RMSD, RMSF, SASA and Rg evaluations graphs. Taken together, it has been concluded that tariquidar predicted exhibited better repositioning profiles as compared to other screened FDA approved drugs and may be use in the treatment of AD after in-vitro and clinical assessment in future.

### Supplementary Information

Supplementary Information.

## Data Availability

The data used or analyzed in the current study are available at different online resources including https://www.uniprot.org/uniprot/Q8WXG6; https://go.drugbank.com/; https://www.dgidb.org/; and http://dsigdb.tanlab.org/DSigDBv1.0/, respectively. All the relevant data has been mentioned in the manuscript whereas, rest of supporting information along with dataset names and accession numbers have been mentioned in the supplementary file.
