# Supplementary material for: Computational prognostic evaluation of Alzheimer’s drugs from FDA-approved database through structural conformational dynamics and drug repositioning approaches
Source: Sci Rep. 2023 Oct 21;13:18022. doi: 10.1038/s41598-023-45347-1 (PMC10590448; doi:10.1038/s41598-023-45347-1)
Supplement: Supplementary file 1 — Supplementary Information. [file 41598_2023_45347_MOESM1_ESM.docx]

**Supplementary data**

**Computational Prognostic evaluation of Alzheimer’s Drugs from FDA-Approved Database through Structural conformational dynamics and Drug Repositioning approaches**

Mubashir Hassan^1^*, Saba Shahzadi^1^, Muhammad Yasir^2^, Wanjoo Chun^2^ Andrzej Kloczkowski^1, 3^*

^1^The Steve and Cindy Rasmussen Institute for Genomic Medicine, Nationwide Children’s Hospital,

Columbus, Ohio 43205, United States; Email: [Mubasher.Hassan@nationwidechildrens.org](mailto:Mubasher.Hassan@nationwidechildrens.org)

^2^Department of Pharmacology, College of Medicine, Kangwon National University, South Korea

^3^Department of Pediatrics, The Ohio State University, Columbus, Ohio 43205, United States; Email: [Andrzej.Kloczkowski@nationwidechildrens.org](mailto:Andrzej.Kloczkowski@nationwidechildrens.org)

**Correspondences**

**Dr. Mubashir Hassan**

The Steve and Cindy Rasmussen Institute for Genomic Medicine, Nationwide Children’s Hospital,

Columbus, Ohio 43205, United States; Email: [Munasher.Hassan@nationwidechildrens.org](mailto:Munasher.Hassan@nationwidechildrens.org)

**Prof. Andrzej Kloczkowski**

The Steve and Cindy Rasmussen Institute for Genomic Medicine, Nationwide Children’s Hospital and Department of Pediatrics, The Ohio State University, Columbus, Ohio 43205, United States; Email: [Andrzej.Kloczkowski@nationwidechildrens.org](mailto:Andrzej.Kloczkowski@nationwidechildrens.org)

**Table S1**. Galaxy refine structure refine models

| **Model** | **GDT-HA** | **RMSD** | **MolProbity** | **Clash score** | **Poor rotamers** | **Rama favored** |
| --- | --- | --- | --- | --- | --- | --- |
| Initial | 1.0000 | 0.000 | 3.809 | 88.4 | 5.7 | 72.4 |
| MODEL 1 | 0.8470 | 0.842 | 2.975 | 48.2 | 1.7 | 85.5 |
| MODEL 2 | 0.8569 | 0.814 | 3.063 | 49.8 | 2.0 | 85.2 |
| MODEL 3 | 0.8526 | 0.827 | 2.901 | 47.8 | 1.4 | 85.8 |
| MODEL 4 | 0.8511 | 0.838 | 2.871 | 49.0 | 1.2 | 85.3 |
| MODEL 5 | 0.8541 | 0.818 | 3.006 | 47.9 | 1.8 | 85.6 |

**Table S2**. Screened drugs against Donepezil from FDA approved library

| **Drug Bank ID** | **Screened Drug Names** | **Similarity Score** |
| --- | --- | --- |
| DB00843 | Donepezil | 1.000 |
| DB07701 | 1-BENZYL-4-[(5 | 0.999 |
| DB13393 | Emetine | 0.837 |
| DB12341 | LY-2456302 | 0.790 |
| DB11324 | Nonivamide | 0.760 |
| DB06774 | Capsaicin | 0.740 |
| DB09120 | Zucapsaicin | 0.731 |
| DB12923 | Gallopamil | 0.643 |
| DB00661 | Verapamil | 0.643 |
| DB14063 | Dexverapamil | 0.629 |
| DB06669 | Arverapamil | 0.613 |
| DB06618 | Cutamesine | 0.579 |
| DB12226 | Terameprocol | 0.547 |
| DB14900 | ISO-1 F-18 | 0.547 |
| DB13865 | Dehydroemetine | 0.532 |
| DB13511 | Clebopride | 0.470 |
| DB12082 | Vesnarinone | 0.461 |
| DB08418 | (4aS | 0.459 |
| DB02929 | K201 free base | 0.448 |
| DB01199 | Tubocurarine | 0.424 |
| DB09083 | Ivabradine | 0.417 |
| DB07738 | N-[1-(5-bromo-2 | 0.383 |
| DB02155 | 3-[(3-sec-butyl-4-hydroxybenzoyl)amino]azepan-4-yl 4-(2-hydroxy-5-methoxybenzoyl)benzoate | 0.370 |
| DB13954 | Estradiol cypionate | 0.345 |
| DB07735 | N-[1-(2 | 0.337 |
| DB04872 | Osanetant | 0.331 |
| DB12408 | PF-03635659 | 0.330 |
| DB13080 | Roluperidone | 0.327 |
| DB14641 | Estriol tripropionate | 0.326 |
| DB13276 | Idanpramine | 0.326 |
| DB04835 | Maraviroc | 0.314 |
| DB00496 | Darifenacin | 0.312 |
| DB02565 | 4-(dimethylamino)-N-[7-(hydroxyamino)-7-oxoheptyl]benzamide | 0.309 |
| DB02505 | N-(R-Carboxy-Ethyl)-Alpha-(S)-(2-Phenylethyl) | 0.303 |
| DB08810 | Cinitapride | 0.294 |
| DB07519 | (6R)-2-amino-6-[2-(3'-methoxybiphenyl-3-yl)ethyl]-3 | 0.275 |
| DB11501 | Bunamidine | 0.275 |
| DB12096 | PF-05175157 | 0.272 |
| DB07567 | 2R | 0.270 |
| DB12853 | DA-6886 | 0.266 |
| DB16182 | CXD101 | 0.266 |
| DB07734 | N-(1-benzylpiperidin-4-yl)-4-sulfanylbutanamide | 0.265 |
| DB11793 | Niraparib | 0.240 |
| DB04881 | Elacridar | 0.234 |
| DB06660 | Saredutant | 0.234 |
| DB08950 | Indoramin | 0.233 |
| DB09286 | Pipamperone | 0.230 |
| DB07237 | {4-[(2R)-pyrrolidin-2-ylmethoxy]phenyl}(4-thiophen-3-ylphenyl)methanone | 0.220 |
| DB06306 | Onalespib | 0.218 |
| DB07147 | methyl (1R | 0.207 |
| DB13310 | Ormeloxifene | 0.206 |
| DB13403 | Oxypertine | 0.205 |
| DB06240 | Tariquidar | 0.203 |
| DB08423 | [5-AMINO-1-(4-FLUOROPHENYL)-1H-PYRAZOL-4-YL][3-(PIPERIDIN-4-YLOXY)PHENYL]METHANONE | 0.197 |
| DB06454 | Sarizotan | 0.191 |
| DB08505 | methyl 4-bromo-N-[8-(hydroxyamino)-8-oxooctanoyl]-L-phenylalaninate | 0.187 |
| DB12837 | UK-500001 | 0.177 |
| DB12731 | Daporinad | 0.177 |
| DB15120 | GSK-239512 | 0.175 |
| DB03944 | 5-[1-(3 | 0.170 |
| DB07642 | 5-{[1-(2-fluorobenzyl)piperidin-4-yl]methoxy}quinazoline-2 | 0.169 |
| DB07279 | N-ETHYL-N-ISOPROPYL-3-METHYL-5-{[(2S)-2-(PYRIDIN-4-YLAMINO)PROPYL]OXY}BENZAMIDE | 0.169 |
| DB15398 | Dihydrocapsiate | 0.166 |
| DB06446 | Dotarizine | 0.166 |
| DB07007 | (3R)-3-[(1 | 0.162 |
| DB11376 | Azaperone | 0.156 |
| DB13790 | Fipexide | 0.156 |
| DB07002 | 4-({4-[(4-methoxypyridin-2-yl)amino]piperidin-1-yl}carbonyl)benzonitrile | 0.156 |
| DB00637 | Astemizole | 0.155 |
| DB15377 | ATB-346 | 0.153 |
| DB08280 | (1S | 0.152 |
| DB05713 | LY-517717 | 0.147 |
| DB06884 | 4-HYDROXY-N'-(4-ISOPROPYLBENZYL)BENZOHYDRAZIDE | 0.147 |
| DB05171 | E-2012 | 0.146 |
| DB12725 | TD-8954 | 0.143 |
| DB07537 | N'-(6-aminopyridin-3-yl)-N-(2-cyclopentylethyl)-4-methyl-benzene-1 | 0.143 |
| DB12867 | Benperidol | 0.142 |
| DB05422 | OPC-14523 | 0.142 |
| DB12886 | GSK-1521498 | 0.141 |
| DB08927 | Amperozide | 0.138 |
| DB01187 | Iophendylate | 0.136 |
| DB06144 | Sertindole | 0.133 |
| DB12981 | XL-888 | 0.133 |
| DB00913 | Anileridine | 0.130 |
| DB16080 | Acolbifene | 0.130 |
| DB13042 | Fenoverine | 0.129 |
| DB03596 | N-[2-(1h-Indol-5-Yl)-Butyl]-4-Sulfamoyl-Benzamide | 0.128 |
| DB01238 | Aripiprazole | 0.124 |
| DB16124 | Chiauranib | 0.124 |
| DB06555 | Siramesine | 0.123 |
| DB00706 | Tamsulosin | 0.121 |
| DB13687 | Niaprazine | 0.119 |
| DB05414 | Pipendoxifene | 0.118 |
| DB12289 | Darexaban | 0.117 |
| DB15688 | Vazegepant | 0.116 |
| DB04842 | Fluspirilene | 0.114 |
| DB07834 | N-(cyclopropylmethyl)-2'-methyl-5'-(5-methyl-1 | 0.114 |
| DB06401 | Bazedoxifene | 0.113 |
| DB11732 | Lasmiditan | 0.112 |
| DB07561 | (2Z)-2-cyano-N-(3'-ethoxybiphenyl-4-yl)-3-hydroxybut-2-enamide | 0.111 |
| DB07643 | 5-{[1-(2 | 0.110 |
| DB08489 | N4-HYDROXY-2-ISOBUTYL-N1-(9-OXO-1 | 0.109 |
| DB07156 | (4Z)-6-bromo-4-({[4-(pyrrolidin-1-ylmethyl)phenyl]amino}methylidene)isoquinoline-1 | 0.107 |
| DB09063 | Ceritinib | 0.106 |
| DB13766 | Lidoflazine | 0.102 |
| DB06077 | Lumateperone | 0.101 |
| DB14813 | PF-232798 | 0.101 |
| DB13520 | Metergoline | 0.099 |
| DB11466 | Tepoxalin | 0.098 |
| DB07878 | N-[(1S)-1-{1-[(1R | 0.098 |
| DB08174 | 5-CHLORO-N-((1R | 0.096 |
| DB08012 | Pirodavir | 0.093 |
| DB01459 | Bezitramide | 0.090 |
| DB08753 | 4-{(1E)-3-OXO-3-[(2-PHENYLETHYL)AMINO]PROP-1-EN-1-YL}-1 | 0.089 |
| DB12361 | Piclozotan | 0.089 |
| DB01745 | N-Alpha-(2-Naphthylsulfonyl)-N(3-Amidino-L-Phenylalaninyl)Isopipecolinic Acid Methyl Ester | 0.086 |
| DB09231 | Benidipine | 0.084 |
| DB02919 | 2 | 0.084 |
| DB06249 | Arzoxifene | 0.083 |
| DB13368 | Motretinide | 0.082 |
| DB06896 | 1-(4-fluorophenyl)-N-[3-fluoro-4-(1H-pyrrolo[2 | 0.082 |
| DB00450 | Droperidol | 0.081 |
| DB13094 | Pruvanserin | 0.081 |
| DB08754 | N-Caffeoyltyramine | 0.080 |
| DB06809 | Plerixafor | 0.079 |
| DB15964 | Dynasore | 0.078 |
| DB14934 | GDC-0927 | 0.078 |
| DB03950 | (S)-N-(3-Indol-1-Yl-2-Methyl-Propyl)-4-Sulfamoyl-Benzamide | 0.075 |
| DB03742 | Compound 4-D | 0.074 |
| DB01737 | Nalpha-(2-Naphthylsulfonylglycyl)-3-Amidino-D | 0.074 |
| DB11804 | AZD-5672 | 0.074 |
| DB02715 | Compound 18 | 0.074 |
| DB12877 | Oxatomide | 0.073 |
| DB02240 | Quinacrine mustard | 0.072 |
| DB07787 | 5-FLUORO-1-[4-(4-PHENYL-3 | 0.072 |
| DB13213 | Butaperazine | 0.071 |
| DB11272 | Methyl undecenoyl leucinate | 0.069 |
| DB09238 | Manidipine | 0.069 |
| DB12998 | PF-00217830 | 0.069 |
| DB05294 | Vandetanib | 0.068 |
| DB07811 | N-cyclopropyl-2' | 0.068 |
| DB12799 | Laniquidar | 0.068 |
| DB05476 | WX-UK1 | 0.068 |
| DB15137 | VK-2809 | 0.068 |
| DB12262 | CRS-3123 | 0.067 |
| DB12457 | Rimegepant | 0.066 |
| DB12756 | TAK-901 | 0.066 |
| DB06638 | Quarfloxin | 0.066 |
| DB15442 | Trilaciclib | 0.065 |
| DB15192 | ABT-288 | 0.065 |
| DB04125 | N-Alpha-(2-Naphthylsulfonyl)-N(3-Amidino-L-Phenylalaninyl)-4-Acetyl-Piperazine | 0.064 |
| DB02615 | Compound 19 | 0.064 |
| DB13729 | Camostat | 0.063 |
| DB01640 | 6-(4-{[2-(3-iodobenzyl)-3-oxocyclohex-1-en-1-yl]amino}phenyl)-5-methyl-4 | 0.063 |
| DB07456 | 3-(1H-indol-3-yl)-4-(1-{2-[(2S)-1-methylpyrrolidinyl]ethyl}-1H-indol-3-yl)-1H-pyrrole-2 | 0.063 |
| DB12432 | CC-401 | 0.063 |
| DB15269 | ALK-4290 | 0.062 |
| DB09239 | Niguldipine | 0.062 |
| DB01337 | Pancuronium | 0.062 |
| DB06666 | Lixivaptan | 0.061 |
| DB01691 | Indole Naphthyridinone | 0.061 |
| DB11973 | Tesevatinib | 0.060 |
| DB06486 | Enzastaurin | 0.059 |
| DB06682 | Naproxcinod | 0.059 |
| DB09128 | Brexpiprazole | 0.058 |
| DB03571 | 3-(5-amino-7-hydroxy-[1 | 0.058 |
| DB03549 | Biotinyl P-Nitroaniline | 0.057 |
| DB12114 | Poziotinib | 0.057 |
| DB13032 | Enecadin | 0.056 |
| DB01157 | Trimetrexate | 0.056 |
| DB01950 | AR-AO-14418 | 0.056 |
| DB01482 | Fenethylline | 0.055 |
| DB16218 | Lerociclib | 0.055 |
| DB07458 | 3-(1H-indol-3-yl)-4-{1-[2-(1-methylpyrrolidin-2-yl)ethyl]-1H-indol-3-yl}-1H-pyrrole-2 | 0.054 |
| DB07941 | PH-797804 | 0.054 |
| DB09030 | Vorapaxar | 0.054 |
| DB12686 | PHA-793887 | 0.053 |
| DB12051 | Setrobuvir | 0.052 |
| DB12264 | Atevirdine | 0.052 |
| DB11730 | Ribociclib | 0.052 |
| DB06971 | N-{2-[(4'-CYANO-1 | 0.051 |
| DB06229 | Ocaperidone | 0.050 |
| DB04834 | Rapacuronium | 0.050 |
| DB08922 | Perospirone | 0.050 |
| DB16253 | Nemiralisib | 0.049 |
| DB04772 | 1-GUANIDINO-4-(N-PHENYLMETHANESULFONYL-L-LEUCYL-L-PROLYLAMINO)BUTANE | 0.049 |
| DB12661 | Urapidil | 0.049 |
| DB08865 | Crizotinib | 0.048 |
| DB03072 | 2-{3-[4-(4-Fluorophenyl)-3 | 0.048 |
| DB11773 | BMS-903452 | 0.048 |
| DB12776 | E-6005 | 0.048 |
| DB07249 | N-(5-chloro-1 | 0.047 |
| DB02546 | Vorinostat | 0.047 |
| DB06959 | (2S)-1-(3H-Indol-3-yl)-3-{[5-(6-isoquinolinyl)-3-pyridinyl]oxy}-2-propanamine | 0.047 |
| DB14883 | Lorecivivint | 0.046 |
| DB13988 | SB-269970 | 0.046 |
| DB07078 | (3Z)-6-(4-HYDROXY-3-METHOXYPHENYL)-3-(1H-PYRROL-2-YLMETHYLENE)-1 | 0.046 |
| DB12910 | Emicerfont | 0.045 |
| DB12558 | AEE-788 | 0.045 |
| DB06334 | Tucidinostat | 0.044 |
| DB07183 | N-(4-phenoxyphenyl)-2-[(pyridin-4-ylmethyl)amino]nicotinamide | 0.044 |
| DB12260 | Radiprodil | 0.044 |
| DB12673 | ATX-914 | 0.044 |
| DB15638 | Brensocatib | 0.043 |
| DB06855 | 6-fluoro-2-(2-hydroxy-3-isobutoxy-phenyl)-1H-benzoimidazole-5-carboxamidine | 0.043 |
| DB02008 | 1-(2-Fluorobenzyl)-3-Butyl-8-(N-Acetyl-4-Aminobenzyl)-Xanthine | 0.043 |
| DB02006 | Br-Coeleneterazine | 0.042 |
| DB15247 | Vorolanib | 0.042 |
| DB02989 | CRA_10972 | 0.041 |
| DB01988 | 6((S)-3-Benzylpiperazin-1-Yl)-3-(Naphthalen-2-Yl)-4-(Pyridin-4-Yl)Pyrazine | 0.041 |
| DB15003 | PF-06700841 | 0.041 |
| DB15028 | MK-1064 | 0.040 |
| DB11978 | Glasdegib | 0.040 |
| DB09226 | Brilaroxazine | 0.039 |
| DB07204 | (1S)-1-(1H-INDOL-3-YLMETHYL)-2-(2-PYRIDIN-4-YL-[1 | 0.039 |
| DB04970 | Lesopitron | 0.038 |
| DB12833 | Tandospirone | 0.038 |
| DB12854 | BMS-908662 | 0.036 |
| DB16244 | MK-0773 | 0.035 |
| DB06876 | N-{5-[4-(4-METHYLPIPERAZIN-1-YL)PHENYL]-1H-PYRROLO[2 | 0.034 |
| DB00734 | Risperidone | 0.034 |
| DB11816 | Omecamtiv Mecarbil | 0.034 |
| DB08745 | 4-[[(1E)-2-(4-CHLOROPHENYL)ETHENYL]SULFONYL]-1-[[1-(4-PYRIDINYL)-4-PIPERIDINYL]METHYL]PIPERAZINONE | 0.033 |
| DB14966 | Venglustat | 0.033 |
| DB05409 | NCX 701 | 0.033 |
| DB06940 | N-ethyl-4-{[5-(methoxycarbamoyl)-2-methylphenyl]amino}-5-methylpyrrolo[2 | 0.033 |
| DB12693 | Ritanserin | 0.033 |
| DB12108 | Taselisib | 0.033 |
| DB07350 | (2E)-N-hydroxy-3-[1-methyl-4-(phenylacetyl)-1H-pyrrol-2-yl]prop-2-enamide | 0.032 |
| DB15245 | Olorofim | 0.032 |
| DB15254 | RO-5126766 free base | 0.032 |
| DB07665 | N-[2-(carbamimidamidooxy)ethyl]-2-{6-cyano-3-[(2 | 0.032 |
| DB07514 | 3-(2-aminoquinazolin-6-yl)-1-(3 | 0.032 |
| DB00490 | Buspirone | 0.031 |
| DB00246 | Ziprasidone | 0.030 |
| DB08488 | 4-{[(E)-2-(5-CHLOROTHIEN-2-YL)VINYL]SULFONYL}-1-(1H-PYRROLO[3 | 0.030 |
| DB07226 | N-[4-(2-CHLOROPHENYL)-1 | 0.030 |
| DB01184 | Domperidone | 0.030 |
| DB01836 | [4-(6-Chloro-Naphthalene-2-Sulfonyl)-Piperazin-1-Yl]-(3 | 0.029 |
| DB04888 | Bifeprunox | 0.029 |
| DB14993 | Pyrotinib | 0.029 |
| DB12966 | Falnidamol | 0.029 |
| DB09197 | Mepiprazole | 0.028 |
| DB08746 | 1-[[(1E)-2-(4-CHLOROPHENYL)ETHENYL]SULFONYL]-4-[[1-(4-PYRIDINYL)-4-PIPERIDINYL]METHYL]PIPERAZINE | 0.028 |
| DB08494 | S-{2-[(2-chloro-4-sulfamoylphenyl)amino]-2-oxoethyl} 6-methyl-3 | 0.028 |
| DB07832 | 4-{4-[(5-hydroxy-2-methylphenyl)amino]quinolin-7-yl}-1 | 0.028 |
| DB01026 | Ketoconazole | 0.027 |
| DB07251 | N'-(3-CHLORO-4-METHOXY-PHENYL)-N-(3 | 0.027 |
| DB12758 | Atreleuton | 0.027 |
| DB12655 | Patidegib | 0.027 |
| DB05562 | Naluzotan | 0.026 |
| DB15191 | MAX-40279 | 0.026 |
| DB00251 | Terconazole | 0.026 |
| DB13088 | AZD-0424 | 0.026 |
| DB08822 | Azilsartan medoxomil | 0.025 |
| DB15307 | Atabecestat | 0.025 |
| DB07946 | N-[2-({[amino(imino)methyl]amino}oxy)ethyl]-2-{6-chloro-3-[(2 | 0.024 |
| DB12184 | Gepirone | 0.024 |
| DB15151 | RO-6870810 | 0.024 |
| DB06974 | 5-hydroxy-4-(7-methoxy-1 | 0.024 |
| DB13090 | Zidebactam | 0.024 |
| DB08487 | 3-({4-[(6-CHLORO-1-BENZOTHIEN-2-YL)SULFONYL]-2-OXOPIPERAZIN-1-YL}METHYL)BENZENECARBOXIMIDAMIDE | 0.023 |
| DB08495 | 4-({4-[(6-CHLORO-1-BENZOTHIEN-2-YL)SULFONYL]-2-OXOPIPERAZIN-1-YL}METHYL)BENZENECARBOXIMIDAMIDE | 0.022 |
| DB00590 | Doxazosin | 0.022 |
| DB15169 | H3B-6527 | 0.022 |
| DB04607 | PHENYLAMINOIMIDAZO(1 | 0.021 |
| DB05263 | Caprospinol | 0.021 |
| DB07124 | (2S)-1-(6H-INDOL-3-YL)-3-{[5-(7H-PYRAZOLO[3 | 0.021 |
| DB13966 | Isopropyl myristate | 0.021 |
| DB03878 | N-[4-Methyl-3-[[4-(3-Pyridinyl)-2-Pyrimidinyl]Amino]Phenyl]-3-Pyridinecarboxamide | 0.021 |
| DB12377 | Relebactam | 0.020 |
| DB07049 | (2R)-1-[(4-tert-butylphenyl)sulfonyl]-2-methyl-4-(4-nitrophenyl)piperazine | 0.020 |
| DB08887 | Icosapent ethyl | 0.018 |
| DB04452 | Aminoquinuride | 0.016 |
| DB14666 | Oleoyl chloride | 0.015 |
| DB12382 | R-306465 | 0.013 |
| DB08326 | 2-(6-HYDROXY-1 | 0.013 |
| DB15308 | Ridinilazole | 0.012 |
| DB02465 | Methoxy arachidonyl fluorophosphonate | 0.010 |

**Table S3**. Screened drugs against galantamine from FDA approved library

| **DrugBank** | **Drugs** | **Similarity Score** |
| --- | --- | --- |
| DB00674 | Galantamine | 0.999 |
| DB00318 | Codeine | 0.979 |
| DB01466 | Ethylmorphine | 0.977 |
| DB11490 | Nalorphine | 0.974 |
| DB00295 | Morphine | 0.970 |
| DB01497 | Etorphine | 0.961 |
| DB01480 | Cyprenorphine | 0.957 |
| DB09209 | Pholcodine | 0.951 |
| DB01477 | Codeine methylbromide | 0.950 |
| DB01469 | Acetorphine | 0.946 |
| DB01573 | Benzylmorphine | 0.945 |
| DB01551 | Dihydrocodeine | 0.922 |
| DB00844 | Nalbuphine | 0.910 |
| DB01565 | Dihydromorphine | 0.910 |
| DB01450 | Dihydroetorphine | 0.902 |
| DB01548 | Diprenorphine | 0.889 |
| DB06230 | Nalmefene | 0.880 |
| DB00497 | Oxycodone | 0.867 |
| DB01547 | Drotebanol | 0.840 |
| DB00921 | Buprenorphine | 0.835 |
| DB01512 | Hydromorphinol | 0.832 |
| DB14945 | Florbenazine F-18 | 0.823 |
| DB01192 | Oxymorphone | 0.814 |
| DB01183 | Naloxone | 0.813 |
| DB00704 | Naltrexone | 0.802 |
| DB04509 | N-Methylnaloxonium | 0.770 |
| DB06444 | Dexanabinol | 0.693 |
| DB13471 | Nalfurafine | 0.637 |
| DB04865 | Omacetaxine mepesuccinate | 0.612 |
| DB06217 | Vernakalant | 0.579 |
| DB12543 | Samidorphan | 0.555 |
| DB15495 | Rocaglamide | 0.553 |
| DB00905 | Bimatoprost | 0.540 |
| DB16351 | Volinanserin | 0.531 |
| DB16100 | Axomadol | 0.521 |
| DB15496 | Didesmethylrocaglamide | 0.514 |
| DB11411 | Fenprostalene | 0.514 |
| DB01210 | Levobunolol | 0.508 |
| DB00908 | Quinidine | 0.505 |
| DB01346 | Quinidine barbiturate | 0.494 |
| DB00468 | Quinine | 0.487 |
| DB16271 | Opiranserin | 0.478 |
| DB15241 | Methylsamidorphan | 0.476 |
| DB00193 | Tramadol | 0.475 |
| DB06578 | Tonabersat | 0.458 |
| DB00424 | Hyoscyamine | 0.445 |
| DB01203 | Nadolol | 0.443 |
| DB04861 | Nebivolol | 0.442 |
| DB09184 | Edivoxetine | 0.440 |
| DB00611 | Butorphanol | 0.436 |
| DB00285 | Venlafaxine | 0.426 |
| DB00572 | Atropine | 0.425 |
| DB14035 | Englitazone | 0.404 |
| DB12057 | ORM-12741 | 0.400 |
| DB15439 | Navoximod | 0.399 |
| DB12608 | Emixustat | 0.393 |
| DB06422 | Ticalopride | 0.379 |
| DB13718 | Hydroquinine | 0.379 |
| DB01359 | Penbutolol | 0.376 |
| DB08952 | Indenolol | 0.374 |
| DB09196 | Lubazodone | 0.371 |
| DB01487 | Embutramide | 0.364 |
| DB15300 | Hydroquinidine | 0.361 |
| DB15096 | 18-methoxycoronaridine | 0.356 |
| DB07374 | Anisomycin | 0.356 |
| DB12464 | Bevenopran | 0.346 |
| DB12884 | Lavoltidine | 0.344 |
| DB00688 | Mycophenolate mofetil | 0.341 |
| DB07258 | (R)-pyridin-4-yl[4-(2-pyrrolidin-1-ylethoxy)phenyl]methanol | 0.340 |
| DB01229 | Paclitaxel | 0.336 |
| DB01505 | Etoxeridine | 0.333 |
| DB05284 | CA4P | 0.333 |
| DB05626 | Retaspimycin | 0.332 |
| DB06700 | Desvenlafaxine | 0.331 |
| DB12708 | Sulprostone | 0.329 |
| DB14881 | Oliceridine | 0.324 |
| DB12179 | Secoisolariciresinol | 0.324 |
| DB12637 | Onapristone | 0.324 |
| DB13559 | Rimiterol | 0.324 |
| DB02205 | (+)-Rutamarin alcohol | 0.323 |
| DB12596 | Combretastatin | 0.322 |
| DB13436 | Droxypropine | 0.319 |
| DB09039 | Eliglustat | 0.314 |
| DB11181 | Homatropine | 0.312 |
| DB00195 | Betaxolol | 0.310 |
| DB00866 | Alprenolol | 0.309 |
| DB09351 | Levobetaxolol | 0.308 |
| DB11711 | Navarixin | 0.305 |
| DB02161 | Hydroxy-Phenyl-Acetic Acid 8-Methyl-8-Aza-Bicyclo[3.2.1]Oct-3-Yl Ester | 0.304 |
| DB16243 | TBA-7371 | 0.302 |
| DB13824 | Enprostil | 0.295 |
| DB00654 | Latanoprost | 0.295 |
| DB00973 | Ezetimibe | 0.295 |
| DB00521 | Carteolol | 0.294 |
| DB11785 | Anisodamine | 0.293 |
| DB13822 | Meprotixol | 0.289 |
| DB12113 | CE-224535 | 0.289 |
| DB05137 | Lobeline | 0.288 |
| DB00224 | Indinavir | 0.288 |
| DB13398 | Oxyfedrine | 0.286 |
| DB06191 | Zosuquidar | 0.285 |
| DB00462 | Methscopolamine bromide | 0.280 |
| DB01253 | Ergometrine | 0.279 |
| DB01464 | Furethidine | 0.272 |
| DB01182 | Propafenone | 0.270 |
| DB05013 | Ingenol mebutate | 0.268 |
| DB02424 | Geldanamycin | 0.259 |
| DB13418 | Moxestrol | 0.258 |
| DB12633 | BMS-184476 | 0.256 |
| DB01295 | Bevantolol | 0.255 |
| DB15954 | Strychnine | 0.253 |
| DB00747 | Scopolamine | 0.252 |
| DB14799 | PF-06821497 | 0.248 |
| DB11454 | Prostalene | 0.248 |
| DB13833 | Methylatropine | 0.247 |
| DB08954 | Ifenprodil | 0.246 |
| DB15578 | Desfesoterodine | 0.246 |
| DB07348 | Brefeldin A | 0.245 |
| DB09201 | Ciglitazone | 0.244 |
| DB05253 | Telapristone acetate | 0.244 |
| DB08347 | 4-{[(2S)-3-(tert-butylamino)-2-hydroxypropyl]oxy}-3H-indole-2-carbonitrile | 0.239 |
| DB13605 | Phenoperidine | 0.239 |
| DB13938 | S-40503 | 0.235 |
| DB04946 | Iloperidone | 0.232 |
| DB06212 | Tolvaptan | 0.231 |
| DB08941 | Isoxsuprine | 0.231 |
| DB11649 | Lersivirine | 0.230 |
| DB06469 | Lestaurtinib | 0.230 |
| DB16028 | Azemiglitazone | 0.230 |
| DB13395 | Eprozinol | 0.225 |
| DB05767 | Andrographolide | 0.225 |
| DB01498 | Alphamethadol | 0.224 |
| DB09080 | Olodaterol | 0.223 |
| DB13141 | Ambroxol acefyllinate | 0.223 |
| DB01426 | Ajmaline | 0.221 |
| DB00247 | Methysergide | 0.221 |
| DB12685 | K-134 | 0.221 |
| DB08068 | N-[4-CHLORO-3-(PYRIDIN-3-YLOXYMETHYL)-PHENYL]-3-FLUORO- | 0.220 |
| DB01179 | Podofilox | 0.220 |
| DB00187 | Esmolol | 0.219 |
| DB05805 | NS-2359 | 0.218 |
| DB13443 | Esatenolol | 0.218 |
| DB00979 | Cyclopentolate | 0.217 |
| DB01267 | Paliperidone | 0.215 |
| DB00332 | Ipratropium | 0.215 |
| DB06446 | Dotarizine | 0.214 |
| DB13555 | Prajmaline | 0.213 |
| DB00598 | Labetalol | 0.213 |
| DB00912 | Repaglinide | 0.211 |
| DB12086 | Oxitropium | 0.210 |
| DB09271 | Cimetropium | 0.210 |
| DB02102 | Mozenavir | 0.209 |
| DB12401 | Bromperidol | 0.209 |
| DB11315 | Methscopolamine | 0.208 |
| DB00393 | Nimodipine | 0.208 |
| DB00502 | Haloperidol | 0.208 |
| DB13825 | Fedrilate | 0.207 |
| DB03383 | CP-320626 | 0.207 |
| DB15109 | Ingenol disoxate | 0.207 |
| DB07981 | 2-[1-(4-chlorobenzoyl)-5-methoxy-2-methyl-1H-indol-3-yl]-n-[(1R)-1-(hydroxymethyl)propyl]acetamide | 0.206 |
| DB05239 | Cobimetinib | 0.204 |
| DB02504 | [3-(1-Benzyl-3-Carbamoylmethyl-2-Methyl-1h-Indol-5-Yloxy)-Propyl-]-Phosphonic Acid | 0.204 |
| DB08059 | Wortmannin | 0.203 |
| DB13023 | IPI-493 | 0.203 |
| DB00725 | Homatropine methylbromide | 0.203 |
| DB07984 | 2-[1-(4-chlorobenzoyl)-5-methoxy-2-methyl-1H-indol-3-yl]-n-[(1S)-1-(hydroxymethyl)propyl]acetamide | 0.202 |
| DB06693 | Mevastatin | 0.201 |
| DB00846 | Flurandrenolide | 0.199 |
| DB00723 | Methoxamine | 0.198 |
| DB01475 | Dioxaphetyl butyrate | 0.197 |
| DB04376 | 13-Acetylphorbol | 0.197 |
| DB12941 | Darolutamide | 0.197 |
| DB12643 | Nelivaptan | 0.196 |
| DB04886 | Calanolide A | 0.196 |
| DB15440 | GSK-3117391 | 0.196 |
| DB12752 | Bucindolol | 0.195 |
| DB13666 | Tiemonium iodide | 0.195 |
| DB12782 | SCH-486757 | 0.194 |
| DB05903 | KOS-1584 | 0.193 |
| DB06152 | Nylidrin | 0.193 |
| DB00353 | Methylergometrine | 0.192 |
| DB09300 | Butylscopolamine | 0.192 |
| DB11958 | Brivanib | 0.191 |
| DB00264 | Metoprolol | 0.191 |
| DB00335 | Atenolol | 0.191 |
| DB00838 | Clocortolone | 0.191 |
| DB05410 | NCX 1022 | 0.190 |
| DB00243 | Ranolazine | 0.189 |
| DB12802 | Picropodophyllin | 0.188 |
| DB11478 | Zeranol | 0.188 |
| DB14221 | Beclomethasone 17-monopropionate | 0.188 |
| DB06814 | Protokylol | 0.187 |
| DB00180 | Flunisolide | 0.187 |
| DB01618 | Molindone | 0.187 |
| DB05134 | Tanespimycin | 0.187 |
| DB12312 | ORG-25435 | 0.186 |
| DB09040 | Efinaconazole | 0.186 |
| DB01260 | Desonide | 0.186 |
| DB00227 | Lovastatin | 0.186 |
| DB12463 | Semagacestat | 0.186 |
| DB09095 | Difluocortolone | 0.185 |
| DB15822 | Pralsetinib | 0.184 |
| DB05938 | Edonerpic | 0.184 |
| DB08037 | MK-0731 | 0.184 |
| DB16011 | Levophencynonate | 0.182 |
| DB13494 | Dimetofrine | 0.182 |
| DB11487 | Dexamethasone isonicotinate | 0.182 |
| DB01232 | Saquinavir | 0.182 |
| DB01933 | 7-Hydroxystaurosporine | 0.182 |
| DB00221 | Isoetharine | 0.182 |
| DB13775 | Tertatolol | 0.182 |
| DB06292 | Dapagliflozin | 0.181 |
| DB14723 | Larotrectinib | 0.181 |
| DB11721 | Mitoglitazone | 0.180 |
| DB07783 | 1-((1R)-1-(HYDROXYMETHYL)-3-{6-[(3-PHENYLPROPANOYL)AMINO]-1H-INDOL-1-YL}PROPYL)-1H-IMIDAZOLE-4-CARBOXAMIDE | 0.179 |
| DB01132 | Pioglitazone | 0.178 |
| DB13554 | Moperone | 0.178 |
| DB04866 | Halofuginone | 0.178 |
| DB15784 | Carmoterol | 0.178 |
| DB12900 | Irdabisant | 0.177 |
| DB11687 | E-6201 | 0.177 |
| DB13488 | Bencyclane | 0.176 |
| DB00641 | Simvastatin | 0.176 |
| DB12927 | Theodrenaline | 0.176 |
| DB12013 | Axelopran | 0.176 |
| DB08808 | Bupranolol | 0.175 |
| DB14544 | Hydrocortisone valerate | 0.175 |
| DB08971 | Fluocortolone | 0.175 |
| DB13239 | Neltenexine | 0.174 |
| DB14540 | Hydrocortisone butyrate | 0.174 |
| DB13762 | Dexrabeprazole | 0.174 |
| DB01129 | Rabeprazole | 0.174 |
| DB01580 | Oxprenolol | 0.173 |
| DB14640 | Isoflupredone acetate | 0.173 |
| DB00547 | Desoximetasone | 0.173 |
| DB12049 | Dasolampanel etibutil | 0.172 |
| DB04938 | Ospemifene | 0.172 |
| DB13203 | Bamifylline | 0.172 |
| DB00220 | Nelfinavir | 0.172 |
| DB12570 | CC-223 | 0.171 |
| DB14634 | Fluprednidene acetate | 0.170 |
| DB08328 | PANTOTHENYL-AMINOETHANOL-11-PIVALIC ACID | 0.170 |
| DB12832 | Prednimustine | 0.169 |
| DB09350 | Piperonyl butoxide | 0.169 |
| DB01222 | Budesonide | 0.168 |
| DB12391 | Sagopilone | 0.168 |
| DB13530 | Mepindolol | 0.168 |
| DB04609 | INHIBITOR Q8467 OF DUPONT MERCK | 0.168 |
| DB08732 | NALPHA-[(BENZYLOXY)CARBONYL]-N-[(1R)-4-HYDROXY-1-METHYL-2-OXOBUTYL]-L-PHENYLALANINAMIDE | 0.167 |
| DB13876 | Brofaromine | 0.167 |
| DB01096 | Oxamniquine | 0.167 |
| DB00591 | Fluocinolone acetonide | 0.166 |
| DB00983 | Formoterol | 0.166 |
| DB04862 | Merimepodib | 0.166 |
| DB02668 | JE-2147 | 0.165 |
| DB14637 | Fluprednisolone acetate | 0.165 |
| DB03572 | FR230513 | 0.164 |
| DB00549 | Zafirlukast | 0.163 |
| DB09004 | Clobutinol | 0.162 |
| DB15257 | Milademetan | 0.162 |
| DB03671 | Digoxigenin | 0.162 |
| DB00809 | Tropicamide | 0.161 |
| DB01873 | Epothilone D | 0.161 |
| DB08970 | Fluprednidene | 0.160 |
| DB00557 | Hydroxyzine | 0.159 |
| DB11805 | Saracatinib | 0.158 |
| DB13491 | Fluperolone | 0.158 |
| DB04177 | Digitoxigenin | 0.158 |
| DB00178 | Ramipril | 0.158 |
| DB06987 | (R)-Atenolol | 0.158 |
| DB11762 | Marizomib | 0.158 |
| DB01234 | Dexamethasone | 0.157 |
| DB06258 | Bimoclomol | 0.157 |
| DB11784 | NRX-1074 | 0.157 |
| DB01274 | Arformoterol | 0.157 |
| DB07943 | SD-0006 | 0.156 |
| DB07543 | (S)-carazolol | 0.155 |
| DB14649 | Dexamethasone acetate | 0.155 |
| DB02096 | FR221647 | 0.154 |
| DB03322 | Dexpropranolol | 0.154 |
| DB13512 | Clefamide | 0.153 |
| DB13564 | Zipeprol | 0.153 |
| DB14941 | BMS-817399 | 0.153 |
| DB05695 | NPS-2143 | 0.152 |
| DB12926 | Cafedrine | 0.152 |
| DB12601 | Sonolisib | 0.152 |
| DB12485 | Pimonidazole | 0.151 |
| DB01384 | Paramethasone | 0.151 |
| DB12770 | Lafutidine | 0.151 |
| DB11991 | Oprozomib | 0.151 |
| DB01214 | Metipranolol | 0.150 |
| DB08596 | 5'-deoxy-5'-piperidin-1-ylthymidine | 0.150 |
| DB12140 | Dilmapimod | 0.150 |
| DB00519 | Trandolapril | 0.150 |
| DB12846 | Reproterol | 0.149 |
| DB12499 | Clascoterone | 0.149 |
| DB00443 | Betamethasone | 0.148 |
| DB08344 | 4-chloro-N-(3-methoxypropyl)-N-[(3S)-1-(2-phenylethyl)piperidin-3-yl]benzamide | 0.148 |
| DB14835 | SUVN-G3031 | 0.148 |
| DB15208 | NOP-1A | 0.148 |
| DB09232 | Cilnidipine | 0.147 |
| DB08590 | 1-(3-HYDROXYPROPYL)-2-[(3-NITROBENZOYL)AMINO]-1H-BENZIMIDAZOL-5-YL PIVALATE | 0.147 |
| DB13019 | Henatinib | 0.147 |
| DB14657 | Paramethasone acetate | 0.147 |
| DB05340 | ATL-2502 | 0.147 |
| DB03044 | Doramapimod | 0.146 |
| DB03220 | FR-234938 | 0.146 |
| DB12713 | Sotagliflozin | 0.146 |
| DB13206 | Bamethan | 0.146 |
| DB06781 | Difluprednate | 0.145 |
| DB00960 | Pindolol | 0.145 |
| DB00288 | Amcinonide | 0.145 |
| DB15566 | Prednisolone acetate | 0.145 |
| DB14539 | Hydrocortisone acetate | 0.144 |
| DB05104 | Asimadoline | 0.144 |
| DB01048 | Abacavir | 0.144 |
| DB06471 | Naxifylline | 0.142 |
| DB08973 | Fluclorolone acetonide | 0.142 |
| DB05884 | HCV-086 | 0.142 |
| DB09076 | Umeclidinium | 0.141 |
| DB14673 | Flurandrenolide acetate | 0.141 |
| DB01366 | Procaterol | 0.141 |
| DB12279 | OBP-801 | 0.141 |
| DB12167 | LY-3023414 | 0.141 |
| DB14662 | Flunisolide acetate | 0.141 |
| DB06257 | HE-2200 | 0.141 |
| DB01409 | Tiotropium | 0.140 |
| DB03370 | FR239087 | 0.140 |
| DB11431 | Moxidectin | 0.140 |
| DB15114 | Vamorolone | 0.139 |
| DB08080 | Latrunculin B | 0.139 |
| DB15999 | Prednisolone caproate | 0.139 |
| DB00240 | Alclometasone | 0.139 |
| DB00687 | Fludrocortisone | 0.138 |
| DB12066 | Orteronel | 0.138 |
| DB07167 | 5-CYANO-FURAN-2-CARBOXYLIC ACID [5-HYDROXYMETHYL-2-(4-METHYL-PIPERIDIN-1-YL)-PHENYL]-AMIDE | 0.138 |
| DB02830 | FR236913 | 0.138 |
| DB01185 | Fluoxymesterone | 0.138 |
| DB02621 | Latrunculin A | 0.138 |
| DB08447 | 3-{3-[(DIMETHYLAMINO)METHYL]-1H-INDOL-7-YL}PROPAN-1-OL | 0.138 |
| DB11522 | Isoflupredone | 0.137 |
| DB16236 | Mitapivat | 0.137 |
| DB11689 | Selumetinib | 0.137 |
| DB01047 | Fluocinonide | 0.137 |
| DB12402 | Pumosetrag | 0.136 |
| DB01408 | Bambuterol | 0.136 |
| DB00663 | Flumethasone | 0.136 |
| DB13040 | Gandotinib | 0.136 |
| DB00612 | Bisoprolol | 0.135 |
| DB04652 | Corticosterone | 0.135 |
| DB14703 | Dexamethasone metasulfobenzoate | 0.134 |

**Table S4.** Screening of FDA approved drugs using donepezil **(100)**

| **Target protein and screened Drugs** | **Binding affinity (Kcal/mol)** | **Target protein and screened Drugs** | **Binding affinity (Kcal/mol)** |
| --- | --- | --- | --- |
| MADD_DB00496 | -8.1 | MADD_DB08280 | -7.9 |
| MADD_DB00637 | -8.4 | MADD_DB08418 | -8.7 |
| MADD_DB00661 | -6.5 | MADD_DB08423 | -8.3 |
| MADD_DB00706 | -6 | MADD_DB08489 | -8.7 |
| MADD_DB00913 | -6.7 | MADD_DB08505 | -6.5 |
| MADD_DB01187 | -5.1 | MADD_DB08810 | -7 |
| MADD_DB01199 | -8.5 | MADD_DB08927 | -7.7 |
| MADD_DB01238 | -7.9 | MADD_DB08950 | -8.6 |
| MADD_DB02155 | -8.7 | MADD_DB09063 | -8 |
| MADD_DB02505 | -7.3 | MADD_DB09083 | -7.5 |
| MADD_DB02565 | -6.5 | MADD_DB09120 | -5.9 |
| MADD_DB02929 | -7.7 | MADD_DB09286 | -6.8 |
| MADD_DB03596 | -7.8 | MADD_DB11324 | -6.2 |
| MADD_DB03944 | -8.1 | MADD_DB11376 | -7.7 |
| MADD_DB04835 | -7.7 | MADD_DB11501 | -6 |
| MADD_DB04842 | -8.5 | MADD_DB11732 | -8.8 |
| MADD_DB04872 | -9.2 | MADD_DB11793 | -8.1 |
| MADD_DB04881 | -10.2 | MADD_DB12082 | -8.2 |
| MADD_DB05171 | -7.9 | MADD_DB12096 | -8.3 |
| MADD_DB05414 | -8.4 | MADD_DB12226 | -6.3 |
| MADD_DB05422 | -7.5 | MADD_DB12289 | -8.6 |
| MADD_DB05713 | -9.1 | MADD_DB12341 | -8.3 |
| MADD_DB06144 | -8.1 | MADD_DB12408 | -8.4 |
| MADD_DB06240 | -10.4 | MADD_DB12731 | -7.8 |
| MADD_DB06306 | -7.9 | MADD_DB12837 | -9.2 |
| MADD_DB06401 | -8.4 | MADD_DB12853 | -6.7 |
| MADD_DB06446 | -8.1 | MADD_DB12867 | -8.3 |
| MADD_DB06454 | -9.2 | MADD_DB12886 | -10.3 |
| MADD_DB06555 | -9.3 | MADD_DB12923 | -6.1 |
| MADD_DB06618 | -6.9 | MADD_DB12981 | -8.1 |
| MADD_DB06669 | -6.9 | MADD_DB13080 | -8 |
| MADD_DB06774 | -5.8 | MADD_DB13276 | -8.8 |
| MADD_DB07002 | -8.5 | MADD_DB13310 | -7.8 |
| MADD_DB07007 | -7.8 | MADD_DB13393 | -7.8 |
| MADD_DB07147 | -7.8 | MADD_DB13403 | -7.9 |
| MADD_DB07156 | -7.8 | MADD_DB13511 | -7.4 |
| MADD_DB07237 | -7.8 | MADD_DB13687 | -7.4 |
| MADD_DB07279 | -6.5 | MADD_DB13766 | -8.8 |
| MADD_DB07519 | -8.2 | MADD_DB13790 | -7.5 |
| MADD_DB07537 | -8.3 | MADD_DB13865 | -7.6 |
| MADD_DB07561 | -7.1 | MADD_DB13954 | -8 |
| MADD_DB07567 | -8.5 | MADD_DB14063 | -5.6 |
| MADD_DB07642 | -8.1 | MADD_DB14641 | -7.3 |
| MADD_DB07643 | -7.9 | MADD_DB14900 | -5.7 |
| MADD_DB07701 | -7.7 | MADD_DB15120 | -8.3 |
| MADD_DB07734 | -6.1 | MADD_DB15377 | -7.7 |
| MADD_DB07735 | -5.7 | MADD_DB15398 | -5.7 |
| MADD_DB07738 | -6.1 | MADD_DB15688 | -11.1 |
| MADD_DB07834 | -9.2 | MADD_DB16080 | -9 |
| MADD_DB16182 | -8.9 | MADD_DB16124 | -9.2 |

*Standard Donepezil showed -7.4 Kcal/mol

**Table S5.** Screening of FDA approved drugs using donepezil **(100)** against MADD

| **Targeted protein and Screened drugs** | **Binding affinity (Kcal/mol)** | **Targeted protein and Screened drugs** | **Binding affinity (Kcal/mol)** |
| --- | --- | --- | --- |
| MADD_DB00193 | -6.1 | MADD_DB06444 | -6.8 |
| MADD_DB00195 | -5.5 | MADD_DB06578 | -7.5 |
| MADD_DB00285 | -5.9 | MADD_DB06700 | -6.2 |
| MADD_DB00295 | -7.1 | MADD_DB07036 | -8.6 |
| MADD_DB00318 | -7.4 | MADD_DB07086 | -6.3 |
| MADD_DB00424 | -7.1 | MADD_DB07195 | -7 |
| MADD_DB00468 | -7.2 | MADD_DB07258 | -6.4 |
| MADD_DB00497 | -7.5 | MADD_DB07374 | -6.1 |
| MADD_DB00521 | -6.7 | MADD_DB07905 | -7.6 |
| MADD_DB00572 | -7 | MADD_DB08952 | -5.9 |
| MADD_DB00611 | -7.3 | MADD_DB09039 | -7 |
| MADD_DB00654 | -6.7 | MADD_DB09184 | -6.7 |
| MADD_DB00688 | -7.2 | MADD_DB09196 | -6.7 |
| MADD_DB00704 | -7.6 | MADD_DB09209 | -7.7 |
| MADD_DB00844 | -7.6 | MADD_DB09351 | -5.9 |
| MADD_DB00866 | -6.2 | MADD_DB11181 | -7 |
| MADD_DB00905 | -7.5 | MADD_DB11411 | -6.9 |
| MADD_DB00908 | -6.9 | MADD_DB11490 | -6.8 |
| MADD_DB00921 | -6.8 | MADD_DB11711 | -7.7 |
| MADD_DB00973 | -7.8 | MADD_DB11785 | -6.4 |
| MADD_DB01183 | -7.2 | MADD_DB12057 | -7.3 |
| MADD_DB01192 | -7.3 | MADD_DB12179 | -6.6 |
| MADD_DB01203 | -6.5 | MADD_DB12464 | -7.5 |
| MADD_DB01210 | -6.6 | MADD_DB12543 | -6.8 |
| MADD_DB01229 | -7.6 | MADD_DB12596 | -6.2 |
| MADD_DB01346 | -7.2 | MADD_DB12608 | -6.3 |
| MADD_DB01359 | -6.2 | MADD_DB12637 | -7.5 |
| MADD_DB01450 | -7.4 | MADD_DB12708 | -6.2 |
| MADD_DB01466 | -7.1 | MADD_DB12884 | -7.4 |
| MADD_DB01469 | -7.2 | MADD_DB13436 | -6.3 |
| MADD_DB01477 | -7 | MADD_DB13471 | -8 |
| MADD_DB01480 | -7 | MADD_DB13559 | -6.3 |
| MADD_DB01487 | -6.2 | MADD_DB13718 | -7.3 |
| MADD_DB01497 | -7.5 | MADD_DB13824 | -6.4 |
| MADD_DB01505 | -5.7 | MADD_DB14035 | -8 |
| MADD_DB01512 | -6.7 | MADD_DB14881 | -7 |
| MADD_DB01547 | -6.2 | MADD_DB14945 | -6.6 |
| MADD_DB01548 | -7.2 | MADD_DB15096 | -6.6 |
| MADD_DB01551 | -7.5 | MADD_DB15241 | -7.3 |
| MADD_DB01565 | -6.7 | MADD_DB15300 | -7.2 |
| MADD_DB01573 | -8.3 | MADD_DB15439 | -7.2 |
| MADD_DB02161 | -7.1 | MADD_DB15495 | -7.2 |
| MADD_DB02205 | -7.1 | MADD_DB15496 | -7.8 |
| MADD_DB04509 | -7.9 | MADD_DB16100 | -6.1 |
| MADD_DB04861 | -7.7 | MADD_DB16243 | -7.7 |
| MADD_DB05284 | -6.6 | MADD_DB16271 | -6.3 |
| MADD_DB05626 | -6.6 | MADD_DB16287 | -7.2 |
| MADD_DB06217 | -7 | MADD_DB16351 | -6.6 |
| MADD_DB06230 | -7.3 | MADD_DB04865 | -7.4 |
| MADD_DB06422 | -5.9 | MADD_DB13822 | -6.3 |
| *Standard galantamine binding affinity (-6.7 Kcal/mol) | | | |


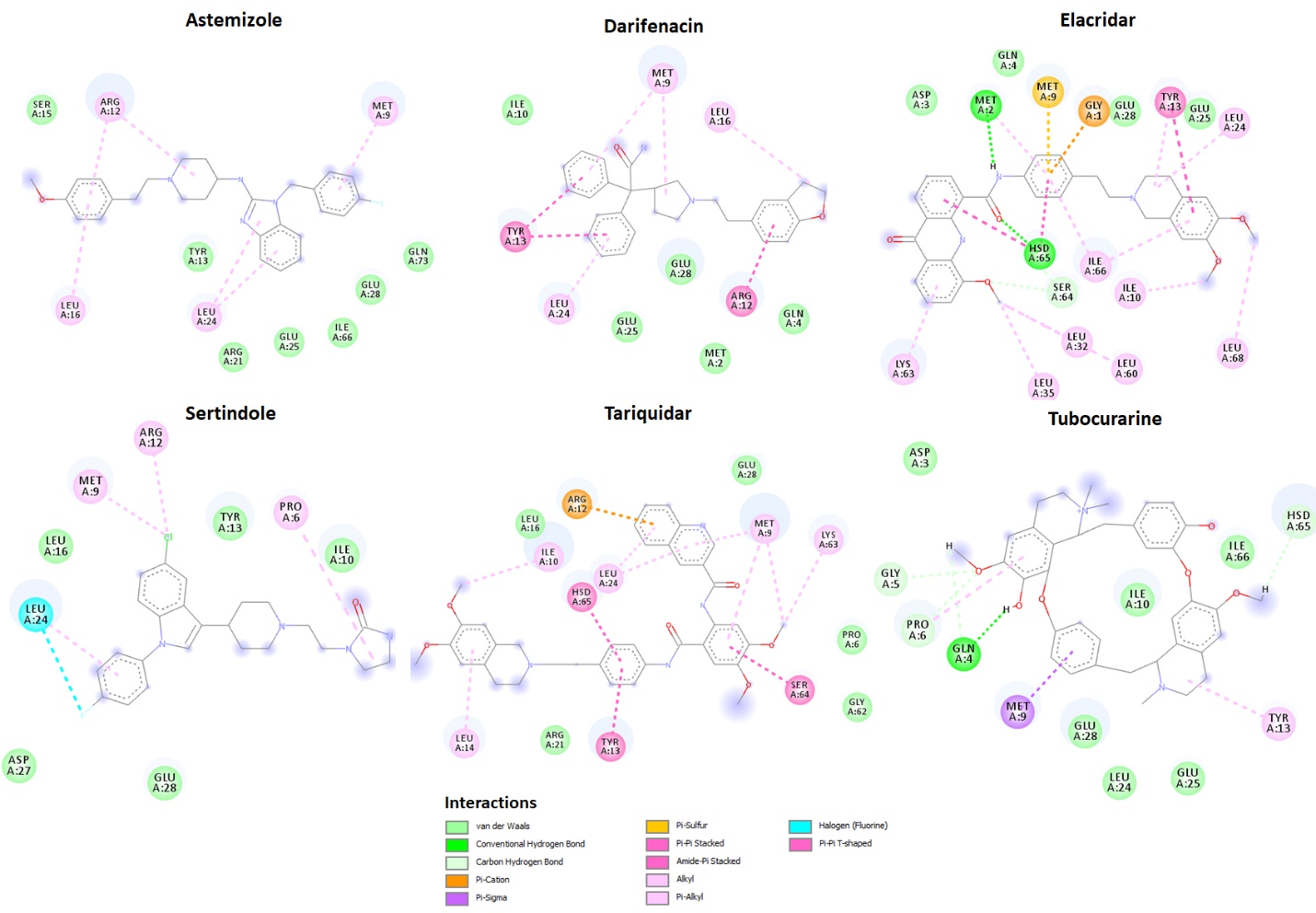


**Figure S6.** Binding pose validation of screened compounds at 100ns MD simulation.
